# ASEAN, Brunei Darussalam, and the Interplay of Foreign Policy and Public Opinion

**DOI:** 10.12688/f1000research.180026.2

**Published:** 2026-09-03

**Authors:** Bama Andika Putra, Sam Rany

**Affiliations:** 1 University of Bristol School of Sociology Politics and International Studies, Bristol, England, UK; 2 Universitas Hasanuddin Fakultas Ilmu Sosial dan Ilmu Politik, Makassar, South Sulawesi, Indonesia; 3 Graduate School, University of Battambang, Krong Battambang, Battambang Province, Cambodia

**Keywords:** Brunei Darussalam, Public Opinion, Foreign Policy, ASEAN, Regionalism

## Abstract

**Background:**

Acknowledging that past studies on the Association of Southeast Asian Nations (ASEAN) have focused on elite and state-centric viewpoints, this study centers on people’s perspectives. The case of Brunei Darussalam, as one of the region’s smallest nations, reveals unique elements in the interaction between public opinion and foreign policy. Given the Sultanate’s consistent stance on closer ties with the regional organization, as evidenced by the positive responses during its ASEAN chairmanship in 2021, do the Brunei public hold similar views?

**Methods:**

To reveal Brunei’s perceptions vis-à-vis ASEAN and explore the relationship between those views and the Sultanate’s ASEAN policies, this qualitative case study examines the applicability of Boucher’s 2024 analytical framework of the ‘top-down’ approach, which argues that the people eventually embrace elites’ views on foreign policy. It utilizes the secondary survey data published in the State of Southeast Asia 2025. It utilizes the survey report published in the State of Southeast Asia 2025.

**Results:**

This study reveals consistency between Brunei respondent’s perceptions with that of the Sultan in the following areas: 1) Measures to enhance ASEAN’s resilience; 2) How ASEAN should respond to the Myanmar Issue and the South China Sea dispute; 3) Questions of alignment amid the great power rivalries influencing the Southeast Asian region; and 4) Perceptions of trust.

**Conclusions:**

The alignment between the Brunei respondents’ perceptions of ASEAN and the Sultan’s implemented policies reveals a unique nexus in shaping public perceptions of foreign policy matters, making Boucher’s top-down approach applicable to the case study.

## 1. Introduction

For decades, the Association of Southeast Asian Nations (ASEAN) has been a cornerstone of Brunei Darussalam’s foreign policy. Brunei officially became the sixth ASEAN member on 07 January 1984, and has since then been active in a number of ASEAN’s regional mechanisms ( MFA, 2026). Despite its small size, Brunei has blended well with the regional organization’s norms, which adhere to the ‘ASEAN Way.’ In the five times that it acted as the ASEAN chair, most recently in 2021, Brunei’s leadership did not yield disappointing responses from fellow ASEAN members ( Bukit & Sworn, 2021, 2022; Chongkittavorn, 2021; IRSEA, 2021; Piri, 2021).

Past discourses on the nexus between Brunei and ASEAN have primarily examined it through the lens of the Brunei elite’s challenges and interests in ASEAN. Following the Brunei ASEAN chair in 2021, for example, the raised theme of ‘We Care, We Prepare, We Prosper’ highlighted Brunei’s unique challenges to respond to the COVID-19 challenge, the military coup in Myanmar, as well as manage the divisions arising from the South China Sea ( Hayat, 2021; IRSEA, 2021; Parameswaran, 2021). As Parameswaran stated, Brunei’s 2021 chair acted as testimony as to how it managed “[…] key foreign relationships, and its capabilities to manage a full plate at home and abroad” ( Parameswaran, 2021). For Brunei, one of the highlights of its challenges was how it would manage relations with China, which, at the time, was approaching the 30th anniversary of China-Brunei bilateral relations. The vast projects and alignment of interests between China and Brunei, marked with the establishment of cooperation in the Sultan Haji Omar Ali Saifuddien Bridge, the Brunei-Guangxi Economic Corridor, and other visible Chinese investment projects in the country, were predicted to cause a dilemma for Brunei elites ( Chongkittavorn, 2021; IRSEA, 2021; Piri, 2021).

Nevertheless, the fear of potentially unfair leadership never materialized. Brunei remained consistent and true to the ASEAN Way during its 2021 ASEAN chairmanship and ensured that its national interests vis-à-vis China did not stand in the way of regional cooperation. Previously, Sultan Hassanal Bolkiah in 2015 stated on the significance of ASEAN for Brunei: “It is hoped that ASEAN can continue to develop a community that is more dynamic, based on the principles that have shaped the region today” ( Yean, 2016, p. 57). Five years later, Brunei stayed true to this by acting as ASEAN chair to counter several of ASEAN’s most challenging circumstances. As Chongkittavorn mentioned, “[…] Brunei epitomizes the true spirit of ASEAN-standing firm and adhering to the founding father’s principles, without compromising ASEAN centrality” ( Chongkittavorn, 2021). As also argued by Bukit and Sworn, analyzing the performance of Brunei’s ASEAN chairmanship in 2021, the exercise of entrepreneurial leadership has allowed Brunei to uphold the significance of ASEAN centrality and ensure that policies taken on behalf of ASEAN are carefully deliberated and formulated not to violate the ASEAN Way ( Bukit & Sworn, 2022).

The success of Brunei’s ASEAN chairmanship in 2021 is a continuation of its success in similar roles in 1989, 1995, 2001, and 2013. It shows that, from the Brunei elite’s perspective, ASEAN is of great importance to Brunei’s foreign policy. However, there are still many elements left unexplored in relation to the discourse of ASEAN’s significance in the eyes of Brunei. More specifically, one can examine whether the Brunei public also holds this favorable perception, or whether the contrary view prevails.

Interestingly, a past study documents some recognition of the lack of exploration of public opinion on ASEAN within the ASEAN member states. In a 2020 study by Lee and Lim, they argued, “[…] most studies on ASEAN take a state-centric and elite-oriented viewpoint” ( Lee & Lim, 2020, p. 803). Against the backdrop of the vast development related to the Brunei and ASEAN relations, this study, therefore, is interested in moving the research agenda in alignment with what Lee and Lim mentioned as “[…] bringing the people of ASEAN into the center of analysis” ( Lee & Lim, 2020, p. 803). Rightfully so, for an absolute monarchy like Brunei, it is difficult to explore the question of public views due to the hierarchical nature of the state system. However, as existing discourses have already made clear the importance of ASEAN to the Brunei elites, an investigation into the public views is also necessary.

In doing so, this qualitative study argues the relevance of the analytical framework introduced in Jean-Christophe Boucher’s 2024 study: ‘Public Opinion, News, Digital Media, and Foreign Policy.’ At the core of the argument, this study emphasizes the importance of public opinion in foreign policy, acknowledging past studies that argue the public makes rational judgments on foreign policy matters ( Aldrich et al., 2006; Conover & Sapiro, 1993; Neuman, 1986; Page, 1996). Therefore, it is pivotal to explore the relationship between public opinion and a state’s foreign policy conduct, delving deeper into the variable of people’s perspectives on pressing foreign policy matters. On this view, Boucher’s ‘top-down’ model, which argues that elites’ views shape the public’s preferences and opinions ( Boucher, 2024), is relevant to Brunei’s public views on ASEAN. This allows a focus on what has been argued in the past: “[…] identifying and measuring the interaction between the public’s foreign policy attitudes and policy elites” ( Boucher, 2024, p. 253).

To claim the consistency between the views of the Brunei elites and the public, this study utilizes the publicly available data from the ‘State of Southeast Asia 2025’ survey report, published by the ISEAS-Yusof Ishak Institute. In this case, the survey includes elites and the informed public, allowing this study to generalize arguments based on the views of those directly involved in the discourse on Brunei and China. The survey provides answers to how Brunei respondents view ASEAN in general, ASEAN’s role in contemporary regional challenges, questions on ASEAN’s potential alignment, and the way forward for the regional organization ( Seah et al., 2025). Using this dataset, this study is able to isolate how Brunei respondents view the regional organization in 2025 and connect this to the potential influence that Brunei elites had in shaping those preferences, based on Brunei’s ASEAN policies during and after its ASEAN chairmanship in 2021. Important to note here is that the respondents in the ISEAS survey represent elements from academia, government, regional organizations, NGOs/media, as well as the private sector, which are more informed populations of Brunei (rather than the overall population). Furthermore, to argue the alignment of views between Brunei’s informed public and the Sultan, policy documents of Brunei, speeches, and general indication of alignment preferences through primary documents are considered in this study.

## 2. Making room for public opinion: A literature review on Brunei in ASEAN

When discussing public opinion in Brunei Darussalam, there are only a few studies directly addressing Brunei’s public opinion on ASEAN. In Yean’s 2016 study, which asked, ‘What does ASEAN mean to ASEAN Peoples?’ Yean’s online survey provided unique insights into how 19–25-year-old respondents view ASEAN awareness, identification as an ASEAN citizen, perception of Brunei’s ASEAN membership, and future concerns about potential challenges encountered by ASEAN (Yean, 2016). Meanwhile, in a broader context, scholars have examined public perceptions of ASEAN member states; however, this excludes Brunei citizens. Benny & Abdullah’s 2011 study, for example, looked at Indonesian citizens’ perceptions of the ASEAN Community ( Benny & Abdullah, 2011). Similarly, Benny undertook a research inquiry into perceptions of the ASEAN Community, examining differences in perceptions among Indonesian, Malaysian, and Singaporean citizens ( Benny, 2015; Benny et al., 2015). The limited number of studies available shows the severely limited understanding of academia regarding the dynamics of Brunei’s public opinion.

Nevertheless, alternative themes can be explored regarding Brunei and ASEAN. Sidelining for the moment the variable of public opinion, several minor discourses still relate to the discussions put forward in this study: Brunei’s placing of ASEAN as its foreign policy cornerstone, Brunei’s use of ASEAN to counter great power rivalries, as well as discussions specific to Brunei’s ASEAN chairmanships. Revealing how existing studies have interpreted and framed Brunei’s foreign policy in relation to ASEAN enables this study to identify potential variables that influence the Brunei respondents’ foreign policy perceptions.

The first discourse focuses the discussion on ASEAN’s role as Brunei’s foreign policy cornerstone. As Weatherbee and Thambipillai argued prior to Brunei’s ASEAN membership in 1984, there were already numerous studies suggesting a potential alignment of interests between the small state of Brunei and the largest regional grouping in Southeast Asia, ASEAN ( Thambipillai, 1982; Weatherbee, 1983). Rightfully so, after Brunei became the sixth ASEAN member, scholars have argued that being integrated into a web of regional cooperation has brought success in advancing Brunei’s economy, security, and generally raised Brunei’s global profile ( Bukit & Sworn, 2022; Druce, 2023; Koh, 2013; Li, 2025; Loon, 2025). More specifically, Mateev’s study showed that a significant advantage for Brunei is the elevation of its status to equal that of the larger ASEAN member states, despite its relatively smaller size ( Mateev, 2022).

Meanwhile, scholars have also interpreted differently the reasons why Brunei has placed great importance on its engagements with ASEAN over the past couple of decades. A 2024 study examined ASEAN’s favorable non-interference policy and how it reflects Brunei’s political identity ( Wei, 2024). Yuhong Li’s 2025 examined the variable of trust, arguing that it could establish greater integration among member states and align interests ( Li, 2025).

Delving deeper into the discourse on Brunei’s relations with ASEAN, several studies have zoomed in on how Brunei perceives ASEAN’s long-standing role in countering great-power rivalries ( Hashim, 2020; Tumala, 2025). Hayat’s 2021 study, for example, argued how ASEAN provides “[…] a unique forum for Brunei to pursue this policy (balancing powers) as a means of stabilizing the regional balance-of-power system in support of a favorable status quo” ( Hayat, 2021). Similarly, Hashim’s 2020 study also stated, “ASEAN became central in managing both intra-Southeast Asian transitions and external relations with great powers” ( Hashim, 2020). These studies acknowledge that the Southeast Asian region is vulnerable to great-power rivalries, as seen in the broader Asia-Pacific context ( Alano, 2023; Ciorciari, 2009; Zha, 2022). Consequently, for smaller states like Brunei (which has a limited capacity to exert influence), engaging closer with ASEAN allows Brunei to actively be involved in the process of managing great power relations directed to cooperative norms and the ASEAN Way in general ( Aminuddin & Purnomo, 2017; Beeson, 2009; Cuyvers, 2019; Tekunan, 2015).

Because of ASEAN’s perceived importance across sectors, Brunei has taken its responsibilities as ASEAN chair seriously. Scholars’ interpretations offer a better understanding of Brunei’s agency in this regional role granted by ASEAN. Bukit and Sworn argued that Brunei exercised “[…] entrepreneurial and intellectual leadership on three key issues confronting the body in 2021” (Myanmar, South China Sea, and COVID-19) ( Bukit & Sworn, 2022, p. 48). Meanwhile, a 2022 study examined the unique tools Brunei used to navigate its chairmanship responsibilities ( Salleh, 2021).

Although existing scholarship documents an evolving discourse seeking to make sense of Brunei’s agency and active participation in ASEAN, the role of public opinion remains unexplored in these studies. Therefore, to better our understanding of how Brunei citizens perceive ASEAN’s importance (and related issues), this study zooms in on Brunei’s perceptions of the regional organization and identifies potential variables that could influence these perceptions. Arguing specifically for a top-down model (alignment between public opinion and elite perceptions/foreign policy conduct), this study will be the first to examine how public opinion is formed in Brunei, measuring the interactive relationship between public opinion and regional foreign policy.

## 3. Public opinion and foreign policy: The proposed analytical framework

For decades, international relations scholars have argued for the significance of public opinion in foreign policy. Within the realism camp, for example, studies (primarily from neoclassical realism) have explored the importance of public opinion in shaping state behaviors ( Schweller, 1998; Waltz, 1979; Wolfers, 1952; Zakaria, 1998). Arguing how public opinions shape the state’s norms, several other studies within the constructivism and English School have argued how public opinions correlate directly to the formation of preferences among the society, including the state’s identity construction ( Browning, 2006; Cantir & Kaarbo, 2012; Finnemore, 2013; Katzenstein, 2003; Steele, 2014; Wehner & Thies, 2014).

Nevertheless, there remains significant debate over the impact of public opinion on foreign policy. By the definition of Key’s 1964 study, public opinion can be understood as “[…] those opinions held by private persons which governments find it prudent to heed” ( Key, 1964, p. 14). Similarly, Boucher argued that public opinion may reflect the particular beliefs, views, and attitudes held by the public ( Boucher, 2024). Therefore, the debate primarily centers on the impact of public opinion, which some have argued is irrational, while others claim it is sound and rational ( Carr, 1936; Holsti, 1996; Lippmann, 1955; Page, 1996; Powlick & Katz, 1998). Without delving into this complex debate, the assertion in this study is limited to understanding the potential relevance of a variable in making sense of contemporary perceptions of a state’s foreign policy.

In doing so, Boucher’s ‘top-down’ model serves as a good starting point to better understand the relations between the Brunei public and foreign policy decisions. As Boucher suggests, there is a clear gap between what political elites know and what the public understands ( Boucher, 2024). Consequently, it is expected that public opinions would use the views of the elites as “[…] a heuristic shortcut for them (the public) to form an opinion” ( Boucher, 2024, p.254). This point has been repeatedly argued by past scholars who fall under the broad category of the top-down model, believing that the interactive relationship between the two variables is best described as consistent with the elite’s views ( Zaller, 1992; Druckman, 2001; Western, 2005; Boucher, 2024).

Bridged to the context of Brunei’s public opinions in matters related to ASEAN, one can conclude that their embraced opinions are consistent with Brunei’s political elites’ view of the regional organization. As perhaps clear by this point, Sultan Hassanal Bolkiah has a favorable perception of ASEAN, and this is evident throughout Brunei’s ASEAN chairmanships. Therefore, the argument proposed is that Brunei’s past active engagements continue to effectively shape policy preferences and public views of Brunei’s external outlook in relation to the Southeast Asian region and ASEAN.

Nevertheless, by looking at the interaction between foreign policy attitudes and the state’s actual foreign policy, one can also infer that an interactive relationship exists between public opinion and the elite, influencing one another. Perhaps out of the three models explored by Boucher, the least possible to be relevant in the case of an absolute monarchy like Brunei is the bottom-down model, in which the people influence the elite’s perception towards foreign policy matters ( Owen, 1994; Moravcsik, 1997; Russett & Oneal, 2001; Bueno de Mesquita et al., 2003; Tomz & Weeks, 2013; Tomz, Weeks & Yarhi-Milo, 2020). However, another body of literature argues that public opinion and the elite’s views influence one another ( Sobel, 2001; Soroka & Wlezien, 2004), described by Boucher as, “A third position (that) tries to reconcile the previous two (bottom-up, top-down models) by observing that political elites and public opinion have developed an interactive relationship, thus influencing each other” ( Boucher, 2024, p.254).

Therefore, it is beyond the scope of this study to assert which model would be most favorable for explaining the relationship between public opinion and foreign policy in the context of Brunei vis-à-vis ASEAN. The discussions, therefore, will first focus on explaining how the top-down model is most likely the one that allows the public to embrace certain perceptions. However, it will also acknowledge the potential for citizens’ views to influence the Brunei elite’s perceptions of ASEAN. The arguments presented in this article also consider the highly restricted nature of Brunei’s information flow, in which accessible information would tend to be based on the Sultan’s preferences.

In doing so, this discussion section is structured as follows. First, it aims to reveal how the Bruneian people perceive the significance of ASEAN across different questions and elements related to Southeast Asian regional dynamics. Understanding Brunei’s public perceptions is done through the use of published data from the State of Southeast Asia survey report 2025, specifically focusing on Brunei respondents’ answers. Several questions focus on Brunei respondents’ perceptions of ASEAN amid international developments, how ASEAN responds to major powers’ regional influence, and ASEAN’s position in the US-China rivalry. After that, it offers a top-down model that focuses on the thesis that Brunei’s elites’ foreign policy preferences are aligned with the perceptions of the Brunei respondents, primarily through events during Brunei’s ASEAN chairmanship in 2021. Elite perceptions, in the context of Brunei, are defined as the policies undertaken by the nation as a whole, without distinguishing between those of different ministries. As an absolute monarchy, the expectation is that differences of opinion are near non-existent, and that Sultan Hassanal Bolkiah is the driving force of how Brunei should act in relation to ASEAN.

## 4. How Brunei citizens view ASEAN

There is no doubt that Brunei elites view ASEAN with great importance; however, does a similar perception exist among the Brunei people, as represented by the informed public? This section first examines Brunei’s public perceptions, focusing on the dataset of Brunei respondents that this study retrieved from the ISEAS Yusof Ishak Institute’s State of Southeast Asia survey report 2025. After revealing those insights, the analytical framework of Boucher’s interactive relationship between public opinion and foreign policy is adopted to decipher what causes the Brunei respondents to embrace certain perceptions of ASEAN.

### 4.1 Disclosing Brunei Respondents’ Perceptions: Insights from the State of Southeast Asia Survey Report

The State of Southeast Asia 2025 provided readers with an overview of how Southeast Asians viewed particular dynamics in the region. Out of the 2,023 respondents, the total number of respondents from Brunei accumulates to 7.4% ( Seah et al., 2025), which have been selected through what the Yusof Ishak Institute mentions as a “[…] screened on their knowledge of ASEAN and the level of interest in current affairs” ( Seah et al., 2025, p. 3). Therefore, through the online mixed purposive sampling method employed, the State of Southeast Asia survey report in 2025 focused on respondents from several specific affiliation categories: academia, government officials, regional organizational personnel, non-government organizations/media, and the private sector ( Seah et al., 2025).

The Brunei respondents’ views on ASEAN can be divided into several specific sectors. The first sector asks the Brunei respondents for their opinions on certain international developments related to ASEAN. When asked, “what is your top concern about ASEAN,” as seen in Table 1 below, Brunei respondents mainly expressed concerns over the regional organization itself lacking the capacity to be relevant in the new world order (30.9%), followed by an external-based challenge that relates to ASEAN being an “[…] arena of major power competition and its member states may become major power proxies” (26.2%) ( Seah et al., 2025, p.16). Meanwhile, an equal number of votes chose the concerns of disunity (13.4%), disconnection from ordinary people (16.8%), and the inability to return to pre-pandemic economic growth rates (12.8%). In relation to that question, when asked how ASEAN can enhance its resilience, the majority of Brunei respondents selected accelerating measures for intra-regional trade and investment (28.2%), followed by the need to cooperate with strategic partners (24.8%) ( Seah et al., 2025).

**Table 1.** Brunei respondents’ answers on the question of what ASEAN’s top concerns are (respondents choose one response).

Questions	The state of Southeast Asia 2025 survey report
“ASEAN is becoming an arena of major power competition, and its member states may become major power proxies.”	26.2%
“ASEAN is becoming increasingly disunited.”	13.4%
“ASEAN is elitist and disconnected from ordinary people.”	16.8%
“ASEAN is slow and ineffective, and thus cannot cope with fluid political and economic developments, and is becoming irrelevant in the new world order.”	30.9%
“ASEAN is unable to return to pre-pandemic economic growth.”	12.8%

Source: Adapted from the State of Southeast Asia 2025 ( Seah et al., 2025).

Equally important is how Brunei respondents viewed several of ASEAN’s pressing issues, such as the challenge in Myanmar, the admission of Timor-Leste, and the South China Sea. Respondents in 2025, in regards to the Myanmar challenge ( Alexandra, 2024; Drajat, 2022; Haacke, 2008; Maizland, 2022; Vasisht, 2024), voted so that ASEAN should “engage in independent dialogue with all key stakeholders, including the National Unity Government (NUG) in Myanmar to build trust” (26.2%) ( Seah et al., 2025, p. 16). Meanwhile, the majority of Brunei respondents (51.7%) voted that the positive developments of Timor-Leste, which in 2025 were greeted with positive signals of being elevated to a full member status ( Cardoso, 2023; Gusmao, 2014; Kammen, 2015; Soares et al., 2024), agreed that the progress should proceed as it is in the status quo. Meanwhile, regarding developments in the South China Sea, two answers stood out, both of which demanded that ASEAN take a firmer stance in the disputed waters. The first is that Brunei respondents asked ASEAN to take a more principled stance in upholding international law (63.1%), and the second is that ASEAN should deter aggressive behavior (54.4%) ( Seah et al., 2025). Similarly, Brunei respondents also favored the currently negotiated Code of Conduct (CoC) (28.9%), embracing the view that the CoC would prevent military activities and energy exploration among the claimant states to the South China Sea ( Seah et al., 2025).

Another group of questions in the State of Southeast Asia survey report 2025 asked Brunei respondents about the major powers’ influence. In one question, they were asked which country or regional organization has the most political and strategic influence in Southeast Asia. Unsurprisingly, the majority of Brunei respondents embraced a positive view towards three leading actors in 2025: ASEAN (18.1%), China (31.5%), and the US (22.8%). When specifically asked about the Brunei respondents’ views on ASEAN, as seen in Table 2 below, a total of 96.3% expressed that they welcomed ASEAN’s “[…] growing regional political and strategic influence,” with only 3.7% choosing that they are worried about ASEAN’s influence ( Seah et al., 2025, p. 35).

**Table 2.** Comparison of the Brunei respondents’ views on ASEAN as a political and strategic power influence in Brunei.

	Choice 1: “I am worried about growing regional political and strategic influence.”	Choice 2: “I welcome its growing regional political and strategic influence.”
Brunei Respondents (2024)	23.8%	76.2%
Brunei Respondents (2025)	3.7%	96.3%

Source: Adapted from the State of Southeast Asia 2025 ( Seah et al., 2025).

Meanwhile, still on the general topic of major power influences in Southeast Asia, Brunei respondents were also asked about their confidence in the state or organization that would champion the global free trade agenda and uphold international law. Regarding the latter question, Brunei respondents ranked China first with 32.2% of the vote, and ASEAN ranked second with 16.1% ( Seah et al., 2025). However, when it comes to the question of the actor providing leadership to maintain and uphold international law, as seen in Table 3 below, ASEAN ranked first with 20.8%, followed by China with 16.8% ( Seah et al., 2025). This shows that Brunei respondents still hold a positive view of ASEAN and are confident that it can provide the leadership needed to uphold a rules-based order.

**Table 3.** Brunei respondents’ perception of the country/organization that offers leadership in maintaining a rules-based order.

	The state of Southeast Asia 2025 survey report (percentage of Brunei respondents choosing this option)
ASEAN	20.8%
Australia	6.0%
China	16.8%
The European Union	13.4%
India	3.4%
Japan	11.4%
Republic of Korea	4.0%%
The United Kingdom	4.7%%
The United States	18.8%

Source: Adapted from the State of Southeast Asia 2025 ( Seah et al., 2025).

One emerging dynamic that ASEAN encounters as of late, is the formation of informal groupings such as the Quad (Quadrilateral Security Dialogue), BRICS (Brazil, Russia, India, China, and South Africa), and AUKUS (Australia, United Kingdom, United States), to name a few ( Rodgers, 2022; Saha, 2018; Sarkar, 2014; Thakur, 2014). The question, therefore, in relation to ASEAN, is how they should respond to the emergence of these informal groupings, which academics in the past have classified as a manifestation of the great power rivalries taking place in the Asia Pacific ( Patrick, 2024; Thakur, 2014; Thies & Nieman, 2017; van Noort, 2019). For example, regarding potential cooperation between ASEAN and the Quad, 32.3% of Brunei respondents voted that it would be beneficial for the Southeast Asian region ( Seah et al., 2025). However, there is a slight inconsistency in how Brunei respondents responded when asked about the emergence of new informal groupings, including what ASEAN should do. The majority of voters expressed caution, by choosing “ASEAN member states should be mindful of joining groupings that risk undermining ASEAN centrality” (27.5%) and “ASEAN should strengthen its convening power and normative influence to ensure its members remain committed and discourage them from joining other groupings” (28.9%) ( Seah et al., 2025, p. 43).

The last section asks respondents for their views on the US-China rivalry and on how ASEAN should respond to the dynamics in Southeast Asia. One question asked is whether ASEAN is currently at the center of the US and China’s influence-seeking efforts in the region; therefore, how should ASEAN respond? A minority of Brunei respondents (8.1%) chose to align ASEAN with one of the major powers ( Seah et al., 2025). Meanwhile, expressing some level of consistency in the need for ASEAN to self-reflect on its capacities, the majority of respondents opted for ASEAN to enhance its resilience and unity (53.0%).

Interestingly, several questions in the 2025 survey report asked about the potential alignment of ASEAN if the regional organization were forced to choose one side. Regarding whom they would prefer to align with between the US and China, the majority of Brunei respondents chose China, with 55.0% of votes ( Seah et al., 2025). Meanwhile, as seen in Table 4 below, in a scenario where ASEAN had to seek the help of a third party to hedge against the US-China strategic rivalry, there was a mix of responses, without a definitive actor the Bruneians perceived as dominant. This is seen in the number of votes for the European Union (24.8%) and Japan (24.8%), which are close to those of other countries, including Australia (13.4%), the United Kingdom (12.8%), and India (16.8%).

**Table 4.** Brunei respondents’ views when asked who ASEAN should seek to as ‘third parties’ to hedge in the face of the US-China great power rivalries.

	The state of Southeast Asia 2024 survey report (percentage of Brunei respondents choosing this option)	The state of Southeast Asia 2025 survey report (percentage of Brunei respondents selecting this option)
Australia	19.5%	13.4%
The European Union	20.8%	24.8%
India	7.8%	16.8%
Japan	31.2%	24.8%
Republic of Korea	6.5%	7.4%
The United Kingdom	14.3%	12.8%

Source: Adapted from the State of Southeast Asia 2025 and 2024 Survey Report ( Seah et al., 2024, 2025).

The data presented in this section show several unique patterns in how Brunei’s public views ASEAN. Within the context of international developments, trust, and the strategic rivalries influencing the Southeast Asian region, there seems to be a strong convergence of views between the respondents and the Sultanate. As will be explored in the next section through Boucher’s top-down analytical framework, it will be shown that Brunei’s public opinion in 2025 converges with the Sultan’s strategic views on ASEAN, expectations, and future projections. This can be seen in the Sultanate’s recent policies, including during Brunei’s ASEAN chairmanship in 2021, which clearly reflected the Sultan’s priorities and views regarding ASEAN.

### 4.2 What influences the perceptions? The role of elite views

To argue that a top-down model reflects the interactive relationship between public opinion and foreign policy, the elite’s views and foreign policy must be highlighted. Therefore, in alignment with the analytical framework of Boucher’s 2024 study, this section will explore the preferences and voices of the Brunei respondents as expressed in the State of Southeast Asia survey report 2025 and argue for consistency between the Brunei respondents’ perceptions and the Sultan’s. However, it also claims there could be room for a combination of a top-down and bottom-up model in Brunei, albeit in a limited sense.

One of the first questions asked of Brunei respondents was what they perceived as the main concerns they had regarding ASEAN. Unsurprisingly, the majority of respondents expressed concern about great power rivalries and the need for ASEAN to enhance its resilience. Nevertheless, on the question of how ASEAN should strengthen its resilience, Brunei respondents voted for ASEAN to focus on increasing intra-regional trade and investment. One way of interpreting this view is the Sultan’s introduction of *Wawasan Brunei 2035*. The grand strategy acknowledges that for too long, Brunei Darussalam has been dependent on its hydrocarbon resources ( Slesman and Baha, 2023; Teja, 2024; CEIC, 2025). Therefore, moving forward, the Sultan is seeking ways to develop sustainable economic measures and diversify the economy ( CSPS, 2022; Koh, 2024; Jetin and Khalid, 2025), a vision eventually embraced by the informed public of Brunei. However, as a small nation of approximately half a million people, Brunei has severely limited choices. Given the Brunei respondents’ embrace of increased intra-regional trade, there is therefore a certain level of consistency between the Brunei respondents’ voices and the Sultan’s approach to diversifying the state’s economy.

Specifically looking at the Brunei respondents’ views on the tensions in Myanmar, what can explain the respondents’ preferences to engage with all actors in Myanmar? Back in 2021, during Brunei’s ASEAN chair, Brunei’s role was pivotal in the conclusion of the ‘Five-Point Consensus.’ The consensus reflected ASEAN’s interests in responding to the Myanmar crisis, calling for an immediate end to violence, acceptance of humanitarian aid, dialogue with all involved conflicting parties, and allowing the ASEAN envoy to visit Myanmar ( Thuzar & Seah, 2022; APHR, 2023; Aung, 2023; Tene, 2024). Sultan Hassanal Bolkiah, at that time, also expressed his willingness to mediate among all parties concerned, which was welcomed by the NUG ( Bandial, 2021). Therefore, when asked four years later about their opinion on how to respond to the Myanmar crisis, the Brunei respondents aligned on the need to engage the NUG to facilitate diverse interests and views in working toward a solution to the situation. Within the same question, only a slight minority answered that ASEAN should not do anything (4.7%) ( Seah et al., 2025), which further shows consistency between the public’s opinions in 2025 being consistent with the Sultan’s ASEAN approach and policies back in 2021. The unique position embraced by the Sultan is related to the vast discourses on issues related to ASEAN’s power limitations, especially within the context of human rights in Southeast Asia.

The Sultan’s South China Sea in the past two decades can also shed light on the Brunei respondents’ perception of how ASEAN should respond to the South China Sea. At first, the results of the 2025 State of Southeast Asia survey may suggest that Bruneians are encouraging a more decisive response from ASEAN. However, a deeper look at the available options would show that the six options themselves lean towards decisive action, albeit in different forms (excluding the option of ASEAN doing nothing) ( Seah et al., 2025). Consequently, the Brunei respondents’ choice to push ASEAN to support actions that deter aggressive behavior and uphold international law remains consistent with the Sultanate’s eventual policies.

As argued by past scholars, for example, Brunei is known to have a somewhat muted response to the South China Sea ( Putra, 2021, 2024; Kurniati, Laksono & Aulia, 2025; Sands, 2026). This is slightly confusing, considering that Brunei is indeed a claimant state in the South China Sea and that intrusions by Chinese law enforcement vessels have occurred in the past ( Elleman, 2017; Hart, 2018; Jacques, 2018; Druce & Julay, 2019). Nevertheless, although this has been the common interpretation, we can also see that the Sultanate is balancing this approach with a more regionally oriented resolution towards the crisis, and one that is centered on the potential role of ASEAN in managing the South China Sea dispute ( Espena & Uy, 2020; Hayat, 2021; IRSEA, 2021). As also argued in the past, ASEAN has the capacity to provide an alternative means to counter issues in the region, which is based on the ASEAN Way. Therefore, although Brunei is a silent claimant state to the South China Sea, it still echoes the importance of maintaining the rule of law in the South China Sea, and undertakes joint exercises with the US as a means to counter potential aggressive behaviors taking place in the disputed waters ( Wilson, 2024; Nguyen, 2025). Therefore, the results of the State of Southeast Asia survey, which reflect the respondent’s perceptions of the nexus between the disputed waters and ASEAN, are consistent with the stance held by the Sultanate.

Similarly, the respondents also expressed confidence in the CoC. This is indeed in alignment with the discourses the Sultanate has disseminated over the past two decades regarding the disputed waters of the South China Sea ( Espena & Uy, 2020; IMOA, 2020; Putra, 2024; Sands, 2026). Brunei believes that it is essential to keep the momentum to conclude the CoC, as it is one of the platforms that have the potential to counter the challenges encountered by claimant states in the South China Sea, and allows for energy exploration deals to take place as a means to reflect the cooperative nature of the seas ( CGTN, 2018; Druce & Julay, 2019; Yilmaz, 2025).

Moving on to the topic of major powers’ regional influence in Southeast Asia, there is also a strong alignment between the views held by the respondents and the actual foreign policy pursued by Brunei years earlier. One perception that may surprise is that the respondents chose China and the US as the strongest political and strategic influences in Southeast Asia, ahead of ASEAN (in third place). Nevertheless, one needs to look more closely at recent trends in Brunei’s bilateral relations with the two countries to understand why the the respondents embraces such a perspective.

With China, the convergence of interests between Brunei and China has been strong. This convergence is primarily marked by the alignment of Brunei’s and China’s grand strategies, namely *Wawasan Brunei 2035* and the Belt and Road Initiative ( Bo, 2017; Koh, 2024; Jetin & Khalid, 2025). The Sultanate has been open to allowing a vast number of Chinese foreign investments into Brunei, whether it be in the infrastructural construction dimensions, or in Brunei’s intentions of diversifying the economy through downstream industries of its oil and gas fields ( CSPS, 2022; Slesman & Baha, 2023; Koh, 2024; Jetin & Khalid, 2025). China has risen in the ranks in terms of its importance to Brunei policymakers, as it is one of the few states openly willing to support the Sultan’s long-term vision. Therefore, it should be no surprise to see alignment between the Sultan’s policies and the eventual perception held by the respondents. As also seen in the question of the survey that asks the Brunei respondents’ confidence in the actor championing the global free trade agenda, the vast export-import cooperation between Brunei and China in the past decade (showing an upward trend) ( WITS, 2025a, 2025b) also explains why respondents opted to choose China first, and ASEAN second. This is critical, considering that in cases such as the ASEAN-China Free Trade Agreement, it is noted that there are legal hurdles and limits to dispute settlement between ASEAN member states and China.

Meanwhile, a similar pattern can be seen with the US. Past studies, for example, have argued for the convergence of national interests between the US and Brunei as of the past several years ( Husseini, 2023; MFA, 2025; USDS, 2024). The only potential contrasting case would be the infamous letter that Trump sent to Sultan Hassanal Bolkiah in 2025, in which Trump expressed harsh words in describing the US and Brunei trade deficits, and warned of a potential increase in export taxes ( Breuninger, 2025). However, despite such an event, there have not been any apparent indications that Brunei is abandoning its relations with the US, as it continues to conduct normal bilateral relations as of the writing of this article. Therefore, it should come as no surprise that Brunei respondents also perceive the US as a formidable political and strategic power in Southeast Asia.

Lastly, one can also draw on the interactive relationship between the respondent’s opinions and the elite’s decided policies and perceptions, based on how the public perceives where ASEAN should position itself amid the US-China strategic rivalry in Southeast Asia. As seen in the 2025 survey report, the majority of respondents chose the option that ASEAN should enhance its capacity to counter pressures from the major powers. The second option was that Brunei respondents chose that ASEAN should not side with China or the US. In the responses, another consistency emerges that aligns with the top-down analytical framework. Some arguments state that Brunei is hedging with the US and China (simultaneously), which shows that Brunei does not wish to side with one power to counter the other ( Husseini, 2023). As the Sultan’s position during the 2021 Brunei chairmanship of ASEAN, the critical part for ASEAN is to enhance its capabilities and make it relevant to Asia Pacific geopolitics through measures as stipulated in the ASEAN Outlook on the Indo-Pacific ( Acharya, 2019; ASEAN, 2019; Singh & Tsjeng, 2020).

These explanations suggest that the elite’s perceptions and policies shape Brunei’s public perceptions. Nevertheless, there is also the potential to argue that an interactive relationship exists between public opinion and foreign policy, in which both variables influence one another ( Sobel, 2001; Soroka & Wlezien, 2004). However, making this conclusion is rather challenging. Brunei Darussalam is an absolute monarchy, which suggests that public opinion’s influence on policymaking is somewhat limited. In another case, it is impossible to make this claim in a closed state like Brunei. Therefore, this study can confidently claim that a top-down framework is applicable for understanding the relations among the variables. Meanwhile, an interactive relationship, in which both variables influence one another, will need to be investigated further in future studies.

## 5. Conclusion

One of the gaps in the study of Brunei Darussalam and ASEAN has been the lack of exploration of public opinion as a variable. Although Brunei is an absolute monarchy ruled by the Sultan, this study shows that some levels of analysis can be applied to understand the nexus of public opinion and foreign policy that suit the unique political context of Brunei. By doing so, it avoids being caught up in the general state-centric, elite-oriented viewpoint and brings citizens’ perspectives to the center of analysis.

In doing so, Boucher’s 2024 analytical framework of the ‘top-down’ model serves this study’s intentions well. In understanding the nexus between public opinion and foreign policy, Boucher’s study argues that elites’ views are consistent with the public’s preferences and opinions. Utilizing data from the 2025 State of Southeast Asia survey report, this study bridges the analytical framework by means of identifying consistency between the Sultanate’s ASEAN policies and perceptions towards the regional organization (primarily during Brunei’s 2021 ASEAN chairmanship), with the public perceptions of Brunei respondents towards ASEAN (seen through respondents’ answers in the 2025 survey).

This study reveals the public opinions of the Brunei people and shows a strong alignment between the Sultan’s past ASEAN perspectives and approaches and the actual perceptions embraced by the respondents. Regarding how Brunei respondents perceived several international developments, for example, Brunei identified that one area it should focus on is intra-regional trade and investment. This means that correlations are made between the Sultan’s recent efforts to diversify Brunei’s economy and how they perceive areas where ASEAN can benefit from further expansion. Furthermore, the Sultan’s 2021 engagements with Myanmar, which led to the eventual ‘Five-Point Consensus’ in the same year, have also been embraced in public perceptions, which called for the establishment of active dialogue with all relevant stakeholders in Myanmar’s internal conflicts, including the NUG.

Similarly, alignment between Brunei’s public opinion and the Sultanate’s ASEAN policy is evident in how they perceive ASEAN’s role in the South China Sea. Given the Sultan’s past approach of adopting a silent claimant status in the disputed waters, public opinion has leaned towards the need for ASEAN to uphold relevant international laws and support actions that deter aggression. These views are consistent with Brunei’s past policies, which, although it has not pursued maritime power projection, have indeed consistently called for ASEAN’s relevance in managing tensions, including through the conclusion of the CoC.

Meanwhile, when Brunei respondents were asked how they viewed the major power contestations in Southeast Asia, they expressed trust in ASEAN and a slight alignment with China. In the question of political and strategic influence, and the actor trusted to champion the global free trade agenda, China claimed the first position from the perspective of the respondents. This is consistent with the Sultan’s foreign policy over the past decade, in which the long-term grand strategy of diversifying the economy has received a positive response from Chinese officials, leading to the convergence of *Wawasan Brunei 2035* and the Belt and Road Initiative. Nevertheless, the importance of ASEAN has remained relevant in both inquiries, ranking significantly higher than those of the other major powers. When respondents were asked what ASEAN should do vis-à-vis the US-China quest for influence, the respondents chose to focus on the within, by enhancing ASEAN’s resilience and unity to counter any pressure from the main powers. This is again consistent with the Sultanate’s foreign policy, which resembles hedging with the US and China, as well as continuing to support ASEAN-introduced measures that would enhance the regional organization’s capacity to address uncertainties to come.

Nevertheless, the analytical framework used to assess Brunei’s foreign policy preferences here comes with its limitations. The most prominent is that the survey reports considered take the views of screened elites and the informed public. Therefore, it does not have the capacity to generalize the overall views of the Brunei public, as it can only argue on the basis of alignment and consistency of views between the respondents who are Bruneian nationals and elites. Second, it also acknowledges that there is room to interpret that the Sultanate’s foreign policy directions are influenced by other factors that are well elaborated within the small state, economic dependence, regional identity, media environment, and regionalization norm literature. However, it is beyond the scope of this article to argue each and every alternative explanation, primarily as this article falls under the category of a case study. These explanations could be an intriguing point of inquiry in future studies.

## Data Availability

The data used for this study can be accessed online:
1. The State of Southeast Asia 2024 Survey Report ( Seah et al., 2024): https://www.iseas.edu.sg/centres/asean-studies-centre/state-of-southeast-asia-survey/the-state-of-southeast-asia-2024-survey-report/
2. The State of Southeast Asia 2025 Survey Report ( Seah et al., 2025): https://www.iseas.edu.sg/centres/asean-studies-centre/state-of-southeast-asia-survey/the-state-of-southeast-asia-2025-survey-report/
