# Supplementary material for: ASEAN, Brunei Darussalam, and the Interplay of Foreign Policy and Public Opinion
Source: F1000Res. 2026 Sep 3;15:633. Originally published 2026 Apr 28. [Version 2] doi: 10.12688/f1000research.180026.2 (PMC13627931; doi:10.12688/f1000research.180026.2)
Supplement: Peer review history

# Reviewer report: Reviewer response for version 2

**DOI:** 10.5256/f1000research.208347.r518463

**Published:** 2026-09-14

**Authors:** Yuni Intarti

**Affiliations:** 1 Universitas Indonesia, Depok, West Java, Indonesia

This manuscript aims to advance research on Brunei and ASEAN by incorporating public opinion into the analysis of Brunei's foreign policy, moving beyond the traditional state-centric and elite-centric perspectives. It employs Boucher's 2024 top-down framework, which posits that elite foreign-policy views can influence the formation of public preferences. The empirical foundation is primarily derived from Brunei-specific responses in the State of Southeast Asia 2025 Survey, supplemented by 2024 survey data and documentary evidence related to Brunei's foreign-policy stances.

 The study investigates respondents' attitudes towards ASEAN resilience and economic cooperation, Myanmar, Timor-Leste, the South China Sea, ASEAN's regional influence, China-US competition, third-party hedging, and trust in regional actors. The authors interpret the alignment between these attitudes and the historical positions or policies of Sultan Hassanal Bolkiah and the Brunei government as indicative of a top-down relationship.

 This research problem is noteworthy. The manuscript is particularly significant in highlighting the challenges of studying public opinion in an absolute monarchy and in linking Brunei studies with the broader Foreign Policy Analysis literature. It also appropriately acknowledges that the ISEAS respondents represent an informed and screened population rather than a representative sample of the general Brunei populace. The survey included 2,023 respondents, with 7.4% from Brunei, selected based on their knowledge of ASEAN and interest in current affairs, and drawn from academia, government, regional organizations, NGOs/media, and the private sector.

 However, several fundamental issues must be addressed to ensure the manuscript's theoretical conclusions are scientifically robust.

**Major issues requiring revision:**

 1. The primary causal assertion surpasses the available evidence. This is the most critical issue and must be addressed. The manuscript demonstrates congruence; however, congruence does not equate to a top-down causal process. For instance, it notes that respondents favor intra-regional trade and investment and interprets this in relation to Wawasan Brunei 2035. Similarly, respondent preferences regarding Myanmar are retrospectively linked to Brunei's conduct during its 2021 ASEAN chairmanship. While these interpretations are plausible, the evidence does not substantiate that respondents formed their attitudes due to the Sultan's preferences.

 This issue becomes particularly evident when the manuscript asserts that elite perceptions and policies 'shape Brunei's public perceptions' and subsequently concludes that the top-down framework can be 'confidently' applied. A cross-sectional survey in 2025, compared retrospectively with policies formulated around or before 2021, cannot independently determine the direction of causality.

 The authors must therefore choose between two strategies. The preferable and realistically achievable solution is to reframe the paper as a study of policy-attitude congruence and systematically replace causal expressions such as shapes, influences, formed by, 'causes,' and 'embraced because of' with is consistent with, corresponds with, converges with, or is compatible with. Alternatively, if the authors wish to maintain a genuine top-down causal argument, they require substantially stronger evidence of the transmission mechanism, such as longitudinal evidence, media or government communication analysis, speeches directed toward domestic audiences, educational/socialization mechanisms, exposure data, or systematic process tracing.

 2. The dependent variable, as currently conceptualized, does not accurately represent 'Brunei public opinion.' This issue must be addressed. The sampling design presents a fundamental construct-validity problem. Respondents were selected based on their knowledge and interest, encompassing government officials, regional-organization personnel, academics, media/NGO representatives, and business respondents. These individuals can be characterized as informed, policy-attentive, or elite-adjacent, but they do not constitute a representative sample of Brunei's population. More critically, some respondents categorized under the 'public' side of the theoretical framework are themselves members of government or policy-oriented elites. This situation introduces potential contamination between the purported independent variable—elite preferences—and the dependent variable—public opinion. Such overlap may artificially enhance the perceived convergence between elite and public views. The authors acknowledge the limitation of representativeness in the conclusion, noting that the survey cannot be generalized to the broader Brunei public. However, this acknowledgment is belated and not consistently reflected throughout the paper. Section headings such as 'How Brunei citizens view ASEAN,"\' along with terms like 'Brunei people' and 'Brunei public opinion, remain overly broad. The terminology should be standardized throughout as 'Brunei respondents,' 'Brunei's informed public,' or 'policy-attentive respondents.' If affiliation-level data are available, a more robust analysis would involve distinguishing between government and non-government respondents. This approach would directly address the theoretical distinction between elite and non-elite attitudes.

 3. The methodology section necessitates a dedicated and significantly more transparent presentation. Currently, methodological details are dispersed across the introduction and analytical sections, rather than being consolidated into a reproducible research design. The article identifies itself as a qualitative case study; however, much of its empirical evidence comprises secondary descriptive survey percentages. Consequently, the manuscript should include a distinct Methods/Research Design section that specifies:

 - the rationale for selecting Brunei as a case study, whether the study is theory-testing, theory-probing, or interpretive.

 - the specific survey materials from 2024 and 2025 utilized.

 - the number of respondents from Brunei.

 - the procedures for survey sampling and weighting.

 - the reasons for selecting particular survey questions. - the operationalization of elite preference.

 - the speeches, government documents, and policy actions that form the elite-policy corpus.

 - and the analytical criteria used to classify survey responses as aligned, partly aligned, or not aligned.

 This is particularly crucial because the paper defines elite perceptions as policies enacted by the nation as a whole and presumes minimal ministerial differences due to the political system. This presumption is an empirical assumption rather than a methodological fact and thus requires justification rather than being inherently integrated into the operationalization.

 4. The analysis is susceptible to confirmation bias, as evidence of correspondence is selected and interpreted post hoc. This issue must be addressed. The manuscript typically commences with a respondent preference and subsequently identifies a government policy that can be broadly interpreted as consistent with this preference. This approach complicates the falsification of the top-down proposition. A more rigorous methodology would involve a transparent analytical matrix, including columns such as: survey question, year, respondent result, predicted elite position, documentary evidence for that elite position, expected direction of congruence, observed congruence, contradictory evidence, and interpretation. Additionally, the paper should examine non-congruent cases rather than solely interpreting convergence. For instance, the discussion on the South China Sea highlights Brunei's traditionally 'muted' stance, while respondents appear to support more decisive ASEAN action. The manuscript subsequently interprets these positions as consistent by adopting a broader rule-of-law framework. While this may ultimately be defensible, the analytical criteria must be established prior to observing the result. Otherwise, almost any position can be reframed as alignment.

 5. Alternative explanations necessitate analytical evaluation rather than mere acknowledgment. The conclusion identifies potential alternative explanations, including small-state characteristics, economic dependence, regional identity, media environment, and regionalization norms. While this inclusion is commendable, relegating these alternatives to future research is inadequate if the article's primary aim is to establish the explanatory value of a specific model. The discussion should, at a minimum, consider whether the observed attitudes are equally consistent with competing explanations. For example, support for ASEAN might stem from small-state security logic, positive attitudes toward China could be attributed to material economic relations, support for hedging might result from structural pressures rather than elite cueing, and ASEAN-oriented attitudes could be a product of long-term regional socialization. Although the authors are not required to test every conceivable theory, they must demonstrate why the evidence more strongly supports the proposed interpretation over obvious alternatives.

 6. Quantitative interpretation requires increased caution and transparency, particularly when percentages are central to the analysis. Although formal inferential statistics are not essential for this qualitative case-study design, the manuscript treats descriptive percentages as substantively meaningful and presents comparisons between the years 2024 and 2025. For instance, Table 4 contrasts the two survey years regarding preferences for third-party hedging. Consequently, the authors should report the actual Brunei sample size for each survey year, rather than merely the 7.4% share of the overall 2025 sample. They should also clarify whether results are weighted, discuss changes in sample composition across survey years, and refrain from interpreting changes between years as substantive shifts unless comparability is established. If inferential significance is not being tested, this should be explicitly stated. Additionally, the term 'correlation' should be avoided unless an actual statistical correlation has been estimated. The conclusion currently asserts that correlations are made between economic diversification and respondents' views. More accurate terms would be correspondence, association at the interpretive level, or descriptive congruence.

 7. Reproducibility is currently incomplete and requires attention. The Data Availability Statement includes links to the 2024 and 2025 ISEAS survey reports, which are beneficial but inadequate for ensuring full reproducibility of the manuscript's analysis. The authors should clearly specify whether they extracted aggregate country percentages or accessed individual-level data. Furthermore, they should provide a supplementary evidence table detailing each survey item utilized, the specific report page or table from which it was sourced, every policy document or speech used as evidence of the Sultanate's position, and the criteria by which the two sets of evidence were compared. In the absence of such an audit trail, while another researcher may reproduce the percentages, they cannot reliably replicate the interpretive finding of 'alignment.'

 The literature review is notably comprehensive, integrating established scholarship on public opinion and foreign policy with recent studies on Brunei, ASEAN, great-power rivalry, and regional developments. The manuscript employs the recent Boucher 2024 framework as its theoretical foundation. Consequently, the age of the literature is not perceived as a significant limitation. Nonetheless, the hierarchy of evidence requires enhancement. Peer-reviewed scholarship and official primary documents should bear the primary analytical responsibility. While media reports and think-tank commentary provide valuable context, they should not replace systematic evidence in determining the Sultanate's policy preferences or the causal mechanisms. Additionally, the abstract contains a statement where the term 'bottom-down model' is used, although 'bottom-up' is evidently intended.

 Finally, the paper addresses a significant question, examines a pertinent empirical case, engages with relatively recent literature, and includes informative descriptive material. Its most robust finding is that the ASEAN-related preferences among informed respondents in Brunei exhibit considerable descriptive alignment with several foreign-policy positions of the Sultanate. This finding alone represents a meaningful contribution. However, the current evidence does not support the stronger assertion that the preferences of the Sultanate's elite caused or influenced these attitudes, nor does it provide a sufficiently rigorous empirical test of Boucher's top-down model. The manuscript should be revised to emphasize this distinction.

Is the case presented with sufficient detail to be useful for teaching or other practitioners?

Yes

Is the work clearly and accurately presented and does it cite the current literature?

Partly

If applicable, is the statistical analysis and its interpretation appropriate?

Partly

Are all the source data underlying the results available to ensure full reproducibility?

Partly

Are the conclusions drawn adequately supported by the results?

Partly

Is the background of the case’s history and progression described in sufficient detail?

Yes

Reviewer Expertise:

ASEAN, border area, regionalism

I confirm that I have read this submission and believe that I have an appropriate level of expertise to confirm that it is of an acceptable scientific standard, however I have significant reservations, as outlined above.

# Response

**Published:** 2026-09-16

**Authors:** bama andika putra

**Affiliations:** University of Bristol School of Sociology Politics and International Studies, Bristol, England, UK

**Comment 1**. The primary causal assertion surpasses the available evidence. This is the most critical issue and must be addressed. The manuscript demonstrates congruence; however, congruence does not equate to a top-down causal process. For instance, it notes that respondents favor intra-regional trade and investment and interprets this in relation to Wawasan Brunei 2035. Similarly, respondent preferences regarding Myanmar are retrospectively linked to Brunei's conduct during its 2021 ASEAN chairmanship. While these interpretations are plausible, the evidence does not substantiate that respondents formed their attitudes due to the Sultan's preferences.

 This issue becomes particularly evident when the manuscript asserts that elite perceptions and policies 'shape Brunei's public perceptions' and subsequently concludes that the top-down framework can be 'confidently' applied. A cross-sectional survey in 2025, compared retrospectively with policies formulated around or before 2021, cannot independently determine the direction of causality.

 The authors must therefore choose between two strategies. The preferable and realistically achievable solution is to reframe the paper as a study of policy-attitude congruence and systematically replace causal expressions such as shapes, influences, formed by, 'causes,' and 'embraced because of' with is consistent with, corresponds with, converges with, or is compatible with. Alternatively, if the authors wish to maintain a genuine top-down causal argument, they require substantially stronger evidence of the transmission mechanism, such as longitudinal evidence, media or government communication analysis, speeches directed toward domestic audiences, educational/socialization mechanisms, exposure data, or systematic process tracing.

 Response: Thank you for this insightful comment. After considering the options, we have went on to proceed with the first strategy choice offered by the reviewer, which is to reframe the paper as a study of policy-attitude congruence. As a consequence, the revisions replaces all causal expressions such as influences, causes, and shapes, and embracements, with several of the following congruence languages: consistent with, coverges with, compatible with, etc. The locations of these revisions can be seen in the following:

 1. Abstract: Background Acknowledging that past studies on the Association of Southeast Asian Nations (ASEAN) have focused on elite and state-centric viewpoints, this study centers on people’s perspectives. The case of Brunei Darussalam, as one of the region’s smallest nations, reveals unique elements in the interaction between public opinion and foreign policy. Given the Sultanate’s consistent stance on closer ties with the regional organization, as evidenced by the positive responses during its ASEAN chairmanship in 2021, do the Brunei public hold similar views? Methods To reveal Brunei’s perceptions vis-à-vis ASEAN and explore the relationship between those views and the Sultanate’s ASEAN policies, this qualitative case study examines the applicability of Boucher’s 2024 analytical framework of the ‘top-down’ approach-under which elite and public positions are expected to converge-to assess the degree of policy-attitude congruence in Brunei. It utilizes the secondary survey data published in the State of Southeast Asia 2025. It utilizes the survey report published in the State of Southeast Asia 2025. Results This study reveals consistency between Brunei respondent’s perceptions with that of the Sultan in the following areas: 1) Measures to enhance ASEAN’s resilience; 2) How ASEAN should respond to the Myanmar Issue and the South China Sea dispute; 3) Questions of alignment amid the great power rivalries influencing the Southeast Asian region; and 4) Perceptions of trust. Conclusions The alignment between the Brunei respondents’ perceptions of ASEAN and the Sultan’s implemented policies reveals a consistent pattern of congruence between elite and public positions on foreign policy matters, a pattern compatible with Boucher’s top-down framework.

 2. Last paragraph of section 1: To claim the consistency between the views of the Brunei elites and the public, this study utilizes the publicly available data from the ‘State of Southeast Asia 2025’ survey report, published by the ISEAS-Yusof Ishak Institute. In this case, the survey includes elites and the informed public, allowing this study to generalize arguments based on the views of those directly involved in the discourse on Brunei and China. The survey provides answers to how Brunei respondents view ASEAN in general, ASEAN’s role in contemporary regional challenges, questions on ASEAN’s potential alignment, and the way forward for the regional organization ( Seah et al., 2025). Using this dataset, this study is able to isolate how Brunei respondents view the regional organization in 2025 and examine the extent to which these preferences are congruent with Brunei’s ASEAN policies during and after its ASEAN chairmanship in 2021. Important to note here is that the respondents in the ISEAS survey represent elements from academia, government, regional organizations, NGOs/media, as well as the private sector, which are more informed populations of Brunei (rather than the overall population). Furthermore, to argue the alignment of views between Brunei’s informed public and the Sultan, policy documents of Brunei, speeches, and general indication of alignment preferences through primary documents are considered in this study.

 3. Paragraph 6 of Section 3: Therefore, it is beyond the scope of this study to assert which model would be most favorable for explaining the relationship between public opinion and foreign policy in the context of Brunei vis-à-vis ASEAN. The discussion will therefore first focus on how the top-down model, understood here as a pattern of policy-attitude congruence rather than demonstrated causal transmission, best explains Brunei respondents’ perceptions. However, it will also acknowledge the potential for citizens’ views to influence the Brunei elite’s perceptions of ASEAN. The arguments presented in this article also consider the highly restricted nature of Brunei’s information flow, in which accessible information would tend to be based on the Sultan’s preferences.

 4. First paragraph of section 4: There is no doubt that Brunei elites view ASEAN with great importance; however, does a similar perception exist among the Brunei people, as represented by the informed public? This section first examines Brunei’s public perceptions, focusing on the dataset of Brunei respondents that this study retrieved from the ISEAS Yusof Ishak Institute’s State of Southeast Asia survey report 2025. After revealing those insights, the analytical framework of Boucher’s interactive relationship between public opinion and foreign policy is adopted to examine the extent to which the Brunei respondents’ perceptions of ASEAN correspond with elite positions.

 5. Title of Section 4.2: Congruence with elite positions: Policy documents and the Sultan’s ASEAN record

 6. Paragraph 2, section 4.2: One of the first questions asked of Brunei respondents was what they perceived as the main concerns they had regarding ASEAN. Unsurprisingly, the majority of respondents expressed concern about great power rivalries and the need for ASEAN to enhance its resilience. Nevertheless, on the question of how ASEAN should strengthen its resilience, Brunei respondents voted for ASEAN to focus on increasing intra-regional trade and investment. One way of interpreting this view is the Sultan’s introduction of *Wawasan Brunei 2035*. The grand strategy acknowledges that for too long, Brunei Darussalam has been dependent on its hydrocarbon resources ( Slesman and Baha, 2023; Teja, 2024; CEIC, 2025). Therefore, moving forward, the Sultan is seeking ways to develop sustainable economic measures and diversify the economy ( CSPS, 2022; Koh, 2024; Jetin and Khalid, 2025), a vision whose economic priorities correspond with those expressed by the informed public of Brunei. However, as a small nation of approximately half a million people, Brunei has severely limited choices. Given the Brunei respondents’ embrace of increased intra-regional trade, there is therefore a certain level of consistency between the Brunei respondents’ voices and the Sultan’s approach to diversifying the state’s economy.

 7. Paragraph 3, section 4.2: Specifically looking at the Brunei respondents’ views on the tensions in Myanmar, how do the respondents’ preferences to engage with all actors in Myanmar correspond with Brunei’s own diplomatic conduct? Back in 2021, during Brunei’s ASEAN chair, Brunei’s role was pivotal in the conclusion of the ‘Five-Point Consensus.’ The consensus reflected ASEAN’s interests in responding to the Myanmar crisis, calling for an immediate end to violence, acceptance of humanitarian aid, dialogue with all involved conflicting parties, and allowing the ASEAN envoy to visit Myanmar ( Thuzar & Seah, 2022; APHR, 2023; Aung, 2023; Tene, 2024). Sultan Hassanal Bolkiah, at that time, also expressed his willingness to mediate among all parties concerned, which was welcomed by the NUG ( Bandial, 2021). Therefore, when asked four years later about their opinion on how to respond to the Myanmar crisis, the Brunei respondents aligned on the need to engage the NUG to facilitate diverse interests and views in working toward a solution to the situation. Within the same question, only a slight minority answered that ASEAN should not do anything (4.7%) ( Seah et al., 2025), which further shows the public’s opinions in 2025 to be consistent with the Sultan’s ASEAN approach and policies back in 2021. The unique position embraced by the Sultan is related to the vast discourses on issues related to ASEAN’s power limitations, especially within the context of human rights in Southeast Asia.

 8. Last paragraph of section 4: These explanations suggest that elite perceptions and policies are consistent with, and precede, the corresponding Brunei public perceptions, though the cross-sectional survey data used here cannot establish the direction of causality between the two. Nevertheless, there is also the potential to argue that an interactive relationship exists between public opinion and foreign policy, in which both variables influence one another ( Sobel, 2001; Soroka & Wlezien, 2004). Testing such a possibility is beyond the scope of the present data. Brunei Darussalam is an absolute monarchy, which suggests that public opinion’s influence on policymaking is likely limited, and it is not possible to verify this claim in a closed state like Brunei with the data available. Consequently, this study argues that a top-down framework of congruence-rather than a demonstrated causal transmission-is the most defensible reading of the relations identified here. Establishing whether elite views actually cause public preferences, and through what mechanism, would require future studies using longitudinal data or a research design on systematic process tracing.

 9. Last paragraph of section 5: Nevertheless, the analytical framework used to assess Brunei’s foreign policy preferences here comes with its limitations. The most prominent is that the survey reports considered take the views of screened elites and the informed public. Therefore, it does not have the capacity to generalize the overall views of the Brunei public, as it can only argue on the basis of alignment and consistency of views between the respondents who are Bruneian nationals and elites. Second, it also acknowledges that there is room to interpret that the Sultanate’s foreign policy directions are influenced by other factors that are well elaborated within the small state, economic dependence, regional identity, media environment, and regionalization norm literature. However, this article is beyond the scope to argue each alternative explanation, primarily because it is a case study. Relatedly, because the survey data are cross-sectional and were collected independently of any measure of respondents’ exposure to elite communication, this study can establish congruence between public and elite positions but cannot, on this evidence alone, establish that elite views caused those public positions; confirming a causal transmission mechanism would require a longitudinal data or process tracing, which we identify as a priority for future research

**Comment 2**: The dependent variable, as currently conceptualized, does not accurately represent 'Brunei public opinion.' This issue must be addressed. The sampling design presents a fundamental construct-validity problem. Respondents were selected based on their knowledge and interest, encompassing government officials, regional-organization personnel, academics, media/NGO representatives, and business respondents. These individuals can be characterized as informed, policy-attentive, or elite-adjacent, but they do not constitute a representative sample of Brunei's population. More critically, some respondents categorized under the 'public' side of the theoretical framework are themselves members of government or policy-oriented elites. This situation introduces potential contamination between the purported independent variable—elite preferences—and the dependent variable—public opinion. Such overlap may artificially enhance the perceived convergence between elite and public views. The authors acknowledge the limitation of representativeness in the conclusion, noting that the survey cannot be generalized to the broader Brunei public. However, this acknowledgment is belated and not consistently reflected throughout the paper. Section headings such as 'How Brunei citizens view ASEAN,"\' along with terms like 'Brunei people' and 'Brunei public opinion, remain overly broad. The terminology should be standardized throughout as 'Brunei respondents,' 'Brunei's informed public,' or 'policy-attentive respondents.' If affiliation-level data are available, a more robust analysis would involve distinguishing between government and non-government respondents. This approach would directly address the theoretical distinction between elite and non-elite attitudes.

 Response: We acknowledge the issue raised by the reviewer, and agree that the terminologies of Brunei public, Brunei people, and Brunei citizens need to be changed to Brunei respondents or Brunei’s informed/policy-attentive public. The revisions covers this concern, in addition to clarifications provided on the respondents and their background occupations. Nevertheless, the revisions provided below does not engage on the reviewer’s suggestion to disaggregate government from non-government respondents, because the survey assessed in this article only publishes aggregate, national-level percentages. The revisions:

 1. First sentences of the abstract: Background Acknowledging that past studies on the Association of Southeast Asian Nations (ASEAN) have focused on elite and state-centric viewpoints, this study centers on people’s perspectives. The case of Brunei Darussalam, as one of the region’s smallest nations, reveals unique elements in the interaction between public opinion and foreign policy. Given the Sultanate’s consistent stance on closer ties with the regional organization, as evidenced by the positive responses during its ASEAN chairmanship in 2021, does Brunei’s informed public hold similar views?

 2. Last paragraph of section 1: Important to note here is that the respondents in the ISEAS survey represent elements from academia, government, regional organizations, NGOs/media, as well as the private sector, which are more informed populations of Brunei (rather than the overall population). Such a limitation is important to acknowledge, as this study frames the respondents as informed public citizens or a policy-attentive public, rather than a general representation of the Brunei people. Furthermore, to argue the alignment of views between Brunei’s informed public and the Sultan, policy documents of Brunei, speeches, and general indications of alignment preferences through primary documents are considered in this study. Accordingly, this study is designed to assess policy-attitude congruence between Brunei’s informed public and the Sultanate’s ASEAN policy record, not to establish a causal transmission-based relationship between the two. This is in consideration of the nature of the ISEAS survey data, which does not make it feasible for inference about the direction of causality.

 3. Last paragraph of section 3: In doing so, this discussion section is structured as follows. First, it aims to reveal how the informed public perceives the significance of ASEAN across different questions and elements related to Southeast Asian regional dynamics. Bruneian people perceive the significance of ASEAN across different questions and elements related to Southeast Asian regional dynamics. Understanding these perceptions is done through the use of published data from the State of Southeast Asia survey report 2025, specifically focusing on Brunei respondents’ answers. Several questions focus on Brunei respondents’ perceptions of ASEAN amid international developments, how ASEAN responds to major powers’ regional influence, and ASEAN’s position in the US-China rivalry. After that, it offers a top-down model that focuses on the thesis that Brunei’s elites’ foreign policy preferences are aligned with the perceptions of the Brunei respondents, primarily through events during Brunei’s ASEAN chairmanship in 2021. Elite perceptions, in the context of Brunei, are defined as the policies undertaken by the nation as a whole, without distinguishing between those of different ministries. As an absolute monarchy, the expectation is that differences of opinion are near non-existent, and that Sultan Hassanal Bolkiah is the driving force of how Brunei should act in relation to ASEAN.

 4. Section title change: 4. How Brunei’s informed public view ASEAN

 5. First two sentences of section 4: There is no doubt that Brunei elites view ASEAN with great importance; however, does a similar perception exist among Brunei’s informed public? This section first examines the perceptions of Brunei respondents retrieved from the ISEAS Yusof Ishak Institute’s State of Southeast Asia survey report 2025.

 6. Last paragraph of section 4.1: The data presented in this section show several unique patterns in how Brunei’s informed public views ASEAN. Amid international developments, trust, and the strategic rivalries shaping Southeast Asia, respondents' views seem to converge strongly with those of the Sultanate. As the next section will show through Boucher’s top-down analytical framework, Brunei’s informed public opinion in 2025 converges with the Sultan’s strategic views on ASEAN, expectations, and future projections. This is evident in the Sultanate’s recent policies, including during Brunei’s ASEAN chairmanship in 2021, which clearly reflected the Sultan’s priorities and views on ASEAN.

 7. Paragraph 10, section 4.2: Lastly, one can also draw on the interactive relationship between the respondents’ opinions and the elite’s decided policies and perceptions, based on how Brunei’s informed public perceives where ASEAN should position itself amid the US-China strategic rivalry in Southeast Asia. As seen in the 2025 survey report, the majority of respondents chose the option that ASEAN should enhance its capacity to counter pressures from the major powers. The second option was that Brunei respondents chose that ASEAN should not side with China or the US. In the responses, another consistency emerges that aligns with the top-down analytical framework. Some arguments state that Brunei is hedging with the US and China (simultaneously), which shows that Brunei does not wish to side with one power to counter the other ( Husseini, 2023). As the Sultan’s position during the 2021 Brunei chairmanship of ASEAN, the critical part for ASEAN is to enhance its capabilities and make it relevant to Asia Pacific geopolitics through measures as stipulated in the ASEAN Outlook on the Indo-Pacific ( Acharya, 2019; ASEAN, 2019; Singh & Tsjeng, 2020).

**Comment 3**: The methodology section necessitates a dedicated and significantly more transparent presentation. Currently, methodological details are dispersed across the introduction and analytical sections, rather than being consolidated into a reproducible research design. The article identifies itself as a qualitative case study; however, much of its empirical evidence comprises secondary descriptive survey percentages. Consequently, the manuscript should include a distinct Methods/Research Design section that specifies:

 - the rationale for selecting Brunei as a case study, whether the study is theory-testing, theory-probing, or interpretive.

 - the specific survey materials from 2024 and 2025 utilized.

 - the number of respondents from Brunei.

 - the procedures for survey sampling and weighting.

 - the reasons for selecting particular survey questions. - the operationalization of elite preference.

 - the speeches, government documents, and policy actions that form the elite-policy corpus.

 - and the analytical criteria used to classify survey responses as aligned, partly aligned, or not aligned.

 This is particularly crucial because the paper defines elite perceptions as policies enacted by the nation as a whole and presumes minimal ministerial differences due to the political system. This presumption is an empirical assumption rather than a methodological fact and thus requires justification rather than being inherently integrated into the operationalization.

 Response: Unfortunately, this isn’t the approach taken by the authors. Although the main data is from survey materials, the ultimate goal is to provide a theory-proving qualitative case study that allows the interpretation of Boucher’s 2024 top-down model to be assessed within the context of Brunei. We did have the option of making a dedicated methodology section, however, we risk using the same materials of what has been published by the survey reports, including a sub-section on case selection, study design, survey materials, sample, weighting, selection of survey questions, etc. Therefore, rather than a dedicated methods section, we focus our third section on the implementation of the framework, not the duplication of the survey’s methods. If there is something that lacks clarity with how we described the research design in the first section, please do inform us.

**Comment 4**: The analysis is susceptible to confirmation bias, as evidence of correspondence is selected and interpreted post hoc. This issue must be addressed. The manuscript typically commences with a respondent preference and subsequently identifies a government policy that can be broadly interpreted as consistent with this preference. This approach complicates the falsification of the top-down proposition. A more rigorous methodology would involve a transparent analytical matrix, including columns such as: survey question, year, respondent result, predicted elite position, documentary evidence for that elite position, expected direction of congruence, observed congruence, contradictory evidence, and interpretation. Additionally, the paper should examine non-congruent cases rather than solely interpreting convergence. For instance, the discussion on the South China Sea highlights Brunei's traditionally 'muted' stance, while respondents appear to support more decisive ASEAN action. The manuscript subsequently interprets these positions as consistent by adopting a broader rule-of-law framework. While this may ultimately be defensible, the analytical criteria must be established prior to observing the result. Otherwise, almost any position can be reframed as alignment.

 Response: We agree with this comment. The revisions first looked to make classification criteria before providing the findings, and made some specific changes on the framing of several case studies:

 1. Paragraph 2, section 4: The classifications of aligned, partly aligned, or not aligned are made in the reading of the survey results. It is considered aligned when a leading response option matches the direction of a specific elite position. Meanwhile, it is considered partially aligned when the elite position diverges in terms of emphasis or degree. Lastly, it is interpreted as not aligned when the leading response option of the informed public opinion contradicts the documented elite position. These indicators are applied consistently across the case studies.

 2, paragraphs 4-5, section 4.2: Brunei’s documented history as a ‘muted’ or ‘silent’ claimant in the South China Sea (2024; Kurniati, Laksono & Aulia, 2025; Sands, 2026) suggests an elite preference for low-key, non-confrontational diplomacy rather than deterrence-oriented rhetoric. This is ofcourse puzzling, considering the recurring intrusions by Chinese law enforcement vessels into Brunei’s claimed waters (Elleman, 2017; Hart, 2018; Jacques, 2018; Druce & Julay, 2019).  The 2025 results only partly match this. Most informed Brunei respondents favored ASEAN taking a more principled stance in upholding international law (63.1%) and deterring aggressive behavior (54.4%). Nevertheless, we note but do not use this to explain the mismatch away, that the survey’s response options were skewed toward decisive framings, which is a measurement limitation that argues for caution rather than a basis for still asserting congruence.

 Respondents’ support for the Code of Conduct (29.8%) is one item that does converge with a specific, sustained element of Brunei’s documented conduct: its continuous diplomatic investment in concluding the CoC ( Espena & Uy, 2020; Hayat, 2021; IRSEA, 2021) and its general disposition to frame the dispute in legal rather than military terms. Brunei’s joint exercises with the US ( Wilson, 2024; Nguyen, 2025) show its conduct is not purely passive, but these exercises are not accompanied by the kind of public deterrence rhetoric the survey’s leading response options imply. That said, the most plausible criteria for classifying the South China Sea case are partly aligned and not aligned. The evidence supports congruence on the narrower claim that Brunei’s informed public and the Sultanate favor a rule-of-law-based, CoC-oriented resolution, but not the broader claim that public and elite convergence exists on how assertively ASEAN or Brunei should act.

 3. paragraph 8 added, section 4.2: Consequently, we categorize the convergence of economic interests as partially aligned rather than fully aligned. The public’s high trust in China is congruent with the economic convergence between the two states’ grand strategies. However, China’s own law enforcement intrusions into Brunei’s claimed waters sit in direct tension with that same public trust.

 4. Section 4.2 (the second last paragraph): Interestingly, several questions in the 2025 survey report asked about the potential alignment of ASEAN if the regional organization were forced to choose one side. Regarding whom they would prefer to align with between the US and China, the majority of Brunei respondents chose China, with 55.0% of votes ( Seah et al., 2025). Meanwhile, as seen in Table 4 below, in a scenario where ASEAN had to seek the help of a third party to hedge against the US-China strategic rivalry, there was a mix of responses, without a definitive actor the Bruneians perceived as dominant. This is reflected in the votes for the European Union (24.8%) and Japan (24.8%), which are close to those for other countries, including Australia (13.4%), the United Kingdom (12.8%), and India (16.8%). Unfortunately, there is no documented Sultanate preference for any specific third party on this question. Therefore, applying the criteria leads to the conclusion that the case is not aligned because it lacks a comparable elite position.

 5. Section 5 (the second last paragraph added): Nevertheless, not every finding in this study supports the top-down reading equally. On the South China Sea and major-power trust concerns, the findings are classified as partly aligned rather than aligned. In addition, third-party hedging is difficult to place within the three specified categories, as no comparable elite position exists.

**Comment 5**: Alternative explanations necessitate analytical evaluation rather than mere acknowledgment. The conclusion identifies potential alternative explanations, including small-state characteristics, economic dependence, regional identity, media environment, and regionalization norms. While this inclusion is commendable, relegating these alternatives to future research is inadequate if the article's primary aim is to establish the explanatory value of a specific model. The discussion should, at a minimum, consider whether the observed attitudes are equally consistent with competing explanations. For example, support for ASEAN might stem from small-state security logic, positive attitudes toward China could be attributed to material economic relations, support for hedging might result from structural pressures rather than elite cueing, and ASEAN-oriented attitudes could be a product of long-term regional socialization. Although the authors are not required to test every conceivable theory, they must demonstrate why the evidence more strongly supports the proposed interpretation over obvious alternatives.

 Response: We agree with this suggestion and have responded to them in the conclusion section. It acknowledges the alternative explanations. The revisions (last two paragraphs of the article):

 The analytical framework used to assess Brunei’s foreign policy preferences here comes with its limitations. The most prominent is that the survey reports considered take the views of screened elites and the informed public. Therefore, it does not have the capacity to generalize the overall views of the Brunei public, as it can only argue on the basis of alignment and consistency of views between the respondents who are Bruneian nationals and elites. Second, it acknowledges that the Sultanate’s foreign policy directions may be influenced by other factors, as elaborated in the literature on small-state economics, economic dependence, regional identity, media environments, and regionalization norms. Although the explanations would not differ significantly, they do provide a different lens of interpretation to assessing Brunei’s foreign policy, which considers different variables utilized in this study.

**Comment 6**: Quantitative interpretation requires increased caution and transparency, particularly when percentages are central to the analysis. Although formal inferential statistics are not essential for this qualitative case-study design, the manuscript treats descriptive percentages as substantively meaningful and presents comparisons between the years 2024 and 2025. For instance, Table 4 contrasts the two survey years regarding preferences for third-party hedging. Consequently, the authors should report the actual Brunei sample size for each survey year, rather than merely the 7.4% share of the overall 2025 sample. They should also clarify whether results are weighted, discuss changes in sample composition across survey years, and refrain from interpreting changes between years as substantive shifts unless comparability is established. If inferential significance is not being tested, this should be explicitly stated. Additionally, the term 'correlation' should be avoided unless an actual statistical correlation has been estimated. The conclusion currently asserts that correlations are made between economic diversification and respondents' views. More accurate terms would be correspondence, association at the interpretive level, or descriptive congruence.

 Response: We agree that the actual Brunei sample sizes and the status of the year-on-year comparisons should be stated plainly. We respond this comment by making two targeted revisions, as seen below:

 1. paragraph before table 2: Another group of questions in the State of Southeast Asia survey report 2025 asked Brunei respondents about the major powers’ influence. In one question, they were asked which country or regional organization has the most political and strategic influence in Southeast Asia. Unsurprisingly, the majority of Brunei respondents embraced a positive view towards three leading actors in 2025: ASEAN (18.1%), China (31.5%), and the US (22.8%). When specifically asked about the Brunei respondents’ views on ASEAN, as seen in Table 2 below, a total of 96.3% of the Brunei respondents surveyed in 2025 expressed that they welcomed ASEAN’s “[…] growing regional political and strategic influence,” with only 3.7% choosing that they are worried about ASEAN’s influence ( Seah et al., 2025, p. 35). Worthy to note is that the 2024-2025 subsample size differs by year, with 78 respondents in 2024 (3.9% of that year’s total), and 150 in 2025 (7.4%). Nevertheless, neither year applies additional weighting within Brunei’s own figures beyond ISEAS’ equal 10% weighting of each country for ASEAN-wide averages.

 2. Paragraph before table 4: Interestingly, several questions in the 2025 survey report asked about the potential alignment of ASEAN if the regional organization were forced to choose one side. Regarding whom they would prefer to align with between the US and China, the majority of Brunei respondents chose China, with 55.0% of votes ( Seah et al., 2025). Meanwhile, as seen in Table 4 below, in a scenario where ASEAN had to seek the help of a third party to hedge against the US-China strategic rivalry, there was a mix of responses, without a definitive actor the Bruneians perceived as dominant. This is reflected in the votes for the European Union (24.8%) and Japan (24.8%), which are close to those for other countries, including Australia (13.4%), the United Kingdom (12.8%), and India (16.8%). As with Table 2, these 2024-2025 figures are reported descriptively, not as a tested or substantive shift. Unfortunately, there is no documented Sultanate preference for any specific third party on this question. Therefore, applying the criteria leads to the conclusion that the case is not aligned because it lacks a comparable elite position.

 3. Paragraph 3, section 5: This study reveals the Brunei respondents and shows a strong alignment between the Sultan’s past ASEAN perspectives and approaches and the actual perceptions embraced by the respondents. Regarding how Brunei respondents perceived several international developments, for example, Brunei identified intra-regional trade and investment as an area it should focus on. This reflects a descriptive correspondence between the Sultan’s recent efforts to diversify Brunei’s economy and how respondents perceive areas where ASEAN can benefit from further expansion, rather than a statistical correlation in the technical sense.   Furthermore, the Sultan’s 2021 engagements with Myanmar, which led to the eventual ‘Five-Point Consensus’ that same year, also resonated in public perceptions, which called for active dialogue with all relevant stakeholders in Myanmar’s internal conflicts, including the NUG.

**Comment 7**: Reproducibility is currently incomplete and requires attention. The Data Availability Statement includes links to the 2024 and 2025 ISEAS survey reports, which are beneficial but inadequate for ensuring full reproducibility of the manuscript's analysis. The authors should clearly specify whether they extracted aggregate country percentages or accessed individual-level data. Furthermore, they should provide a supplementary evidence table detailing each survey item utilized, the specific report page or table from which it was sourced, every policy document or speech used as evidence of the Sultanate's position, and the criteria by which the two sets of evidence were compared. In the absence of such an audit trail, while another researcher may reproduce the percentages, they cannot reliably replicate the interpretive finding of 'alignment.'

 Response: data availability statement revised. However, this section does not permit the display of tables.

 All survey figures used in this study are aggregate, country-level percentages taken from the published reports that can be accessed online:

- **1.** The State of Southeast Asia 2024 Survey Report ( Seah et al., 2024): https://www.iseas.edu.sg/centres/asean-studies-centre/state-of-southeast-asia-survey/the-state-of-southeast-asia-2024-survey-report/
- **2.** The State of Southeast Asia 2025 Survey Report ( Seah et al., 2025): https://www.iseas.edu.sg/centres/asean-studies-centre/state-of-southeast-asia-survey/the-state-of-southeast-asia-2025-survey-report/

**Comment 8**: The literature review is notably comprehensive, integrating established scholarship on public opinion and foreign policy with recent studies on Brunei, ASEAN, great-power rivalry, and regional developments. The manuscript employs the recent Boucher 2024 framework as its theoretical foundation. Consequently, the age of the literature is not perceived as a significant limitation. Nonetheless, the hierarchy of evidence requires enhancement. Peer-reviewed scholarship and official primary documents should bear the primary analytical responsibility. While media reports and think-tank commentary provide valuable context, they should not replace systematic evidence in determining the Sultanate's policy preferences or the causal mechanisms. Additionally, the abstract contains a statement where the term 'bottom-down model' is used, although 'bottom-up' is evidently intended.

 Response: We agree with this comment, and revised the last paragraph of section 3:

 In the Brunei context, elite perceptions are defined as the policies undertaken by the nation as a whole, without distinguishing between different ministries. As an absolute monarchy, the expectation is that differences of opinion are nearly non-existent and that Sultan Hassanal Bolkiah drives how Brunei should act in relation to ASEAN. Therefore, in establishing the Sultanate’s documented positions in ASEAN, the main documents considered are primary sources such as the Sultan’s statements, Brunei government and ASEAN documents, and official policy records. Meanwhile, news reporting and think tank reports are used only to corroborate factual details or provide context and characterizations of events (not as the sole basis for establishing the Sultanate’s policy preferences).

# Reviewer report: Reviewer response for version 2

**DOI:** 10.5256/f1000research.208347.r517510

**Published:** 2026-09-11

**Authors:** Kittisak Wongmahesak

**Affiliations:** 1 North Bangkok University, Bangkok, Bangkok, Thailand

The revised manuscript addresses an important, underexplored question: how public perceptions of ASEAN relate to foreign-policy preferences in Brunei Darussalam, a small, highly centralized Southeast Asian state. Its principal strength is the effort to move beyond conventional elite- and state-centric analyses by examining Bruneian respondents’ views on ASEAN resilience, the Myanmar crisis, the South China Sea, great-power rivalry, and regional trust. The discussion draws on recent survey evidence and relevant literature, and the inclusion of ASEAN Charter constraints and the legal limits of regional decision-making strengthens the institutional context. The article is also clearly structured, accessible, and potentially valuable for scholars of ASEAN, small-state foreign policy, and authoritarian or monarchical political systems.

 Nevertheless, substantial concerns remain before the manuscript can be considered fully scientifically secure. Most importantly, the evidence demonstrates association, thematic consistency, or interpretive convergence, but not that Sultanate policies caused, shaped, or formed respondents’ opinions. Despite the authors’ stated intention to moderate causal claims, expressions such as “effectively shape,” “being formed by,” “what causes,” and “embraced” remain in the revised text. These should be systematically replaced with appropriately cautious formulations, such as “is consistent with,” “may reflect,” or “can plausibly be interpreted as.” The study should not claim to establish the applicability of a top-down model unless it provides evidence of an information-transmission mechanism.

 The methodology also requires further clarification. The manuscript describes itself as a qualitative case study, yet the empirical analysis primarily reproduces aggregate survey percentages and interprets them alongside selected policy statements. The authors should explicitly identify the design as an interpretive case study based on secondary descriptive survey data, specify the criteria for selecting survey questions and official documents, explain how elite preferences were operationalized, and distinguish empirical evidence from authorial interpretation. The screened, policy-attentive respondents cannot represent Brunei’s population generally; therefore, restrict references to “the public” and “Bruneians” to “Bruneian informed respondents.”

 Finally, the manuscript needs a comprehensive editorial audit. The abstract contains methodological duplication, several grammatical and citation inconsistencies remain, and some references appear incomplete or unreliable. The conclusion should be rewritten to match the study's evidentiary limits and engage alternative explanations more substantively, rather than merely deferring them to future research. I recommend approval with reservations, contingent on further substantive revision, with particular attention to causal language, methodological transparency, respondent representativeness, and reference accuracy.

Is the case presented with sufficient detail to be useful for teaching or other practitioners?

Yes

Is the work clearly and accurately presented and does it cite the current literature?

Partly

If applicable, is the statistical analysis and its interpretation appropriate?

Yes

Are all the source data underlying the results available to ensure full reproducibility?

Yes

Are the conclusions drawn adequately supported by the results?

Yes

Is the background of the case’s history and progression described in sufficient detail?

Yes

Reviewer Expertise:

Political Science, Public & Private Management, International Political Economy, Social Research

I confirm that I have read this submission and believe that I have an appropriate level of expertise to confirm that it is of an acceptable scientific standard, however I have significant reservations, as outlined above.

# Reviewer report: Reviewer response for version 1

**DOI:** 10.5256/f1000research.198599.r481188

**Published:** 2026-05-23

**Authors:** Ludiro Madu

**Affiliations:** 1 Universitas Pembangunan Nasional Veteran Yogyakarta, Yogyakarta, Special Region of Yogyakarta, Indonesia

Full report

 The article examines the relationship between public opinion and foreign policy in Brunei Darussalam by focusing on Brunei respondents’ perceptions of ASEAN. Using the State of Southeast Asia 2025 survey and Boucher’s 2024 “top-down” framework, it argues that respondents’ views broadly align with the Sultanate’s ASEAN-oriented foreign policy on ASEAN resilience, the Myanmar issue, the South China Sea, great-power rivalry, and trust. This is a relevant contribution because studies on ASEAN and Brunei remain largely state-centric and elite-oriented, while public opinion in Brunei is still underexplored.

 The article has clear strengths. The topic is original and interesting, as Brunei is rarely central in ASEAN public-opinion studies. The article is generally well-organized, moving from Brunei’s ASEAN membership and chairmanship to the literature, theoretical framework, survey findings, and discussion. It also uses recent literature and relevant survey results, including response patterns on ASEAN’s concerns, Myanmar, Timor-Leste, the South China Sea, major-power influence, and US-China rivalry. The tables on ASEAN’s top concerns and perceptions of political/strategic influence are useful for readers.

 However, I recommend approval with reservations because several issues should be addressed. First, the main concern is the strength of the causal claim. The article shows alignment or consistency between public opinion and elite foreign policy, but the evidence consists mainly of cross-sectional survey data and interpretation of Brunei’s ASEAN policy. This does not fully demonstrate that elite views caused or shaped public opinion. The authors should either soften causal language or provide clearer methodological justification. Terms such as “alignment,” “convergence,” and “consistency” are well supported, whereas “influenced by,” “shaped by,” or “formed by” require more direct evidence of transmission mechanisms.

 Second, the representativeness of the survey data requires clearer discussion. The article notes that the 2025 survey had 2,023 respondents, with Brunei accounting for 7.4%, and that respondents were screened for ASEAN knowledge and interest in current affairs. This means that the data seems to reflect informed or policy-attentive respondents from academia, government, regional organizations, NGOs/media, and the private sector, rather than the entire Brunei population. The authors should, therefore, qualify repeated references to “the Brunei public,” “Brunei people,” and “general public.”

 Third, the methodology should be strengthened. The article describes itself as qualitative, yet much of the analysis relies on descriptive survey percentages. The authors should clarify whether it is a qualitative case study using secondary survey data or a descriptive interpretive analysis. They should also explain how survey questions were selected, how elite views were operationalized, and how policy documents, speeches, or ASEAN chairmanship actions were chosen and interpreted.

 Fourth, the article should more clearly distinguish correlation, interpretation, and evidence. For example, linking respondents’ preference for intra-regional trade and investment to Wawasan Brunei 2035 is plausible, but the article does not directly show that respondents knew or were influenced by that vision. Similarly, claims that public views on Myanmar or the South China Sea were shaped by Brunei’s 2021 ASEAN chairmanship would be stronger with evidence from public discourse, media framing, government communication, speeches, education/socialization, or longitudinal comparison before and after 2021.

 Fifth, alternative explanations should be addressed. Support for ASEAN or preference for hedging may also reflect Brunei’s small-state vulnerability, economic dependence, regional identity, media environment, education, respondents’ professional background, or broader ASEAN norms. The article briefly mentions an interactive relationship between public opinion and elites, but this point remains underdeveloped.

 Sixth, the data availability statement is useful because it links to the 2024 and 2025 State of Southeast Asia survey reports. However, the authors should clarify whether they used only public aggregate tables or country-level raw data. If aggregate percentages were used, this should be stated; if raw data were used, the authors should explain where the dataset can be accessed and how the Brunei subset was isolated.

 Finally, several presentation issues should be corrected. For example, “Brunei respondent’s perceptions” should be “Brunei respondents’ perceptions”; “bottom-down model” appears where “bottom-up model” seems intended; and “soroka wlezien 2004, sobel 2001” is inconsistently formatted. Careful proofreading is needed to improve grammar, consistency, and reference formatting.

 Points that must be addressed to make the article scientifically sound

 1. Clarify that the survey respondents are not necessarily representative of the entire Brunei population and revise broad claims about “the Brunei public” or “Brunei people.”

 2. Soften or justify causal claims about elite influence, since the current evidence supports alignment or consistency more strongly than direct causation.

 3. Expand the methodology section by explaining the research design, survey-question selection, operationalization of “elite views,” and analytical procedure connecting public opinion with foreign policy.

 4. Clarify the data source and reproducibility status, especially whether the study uses aggregate results or raw country-level data.

 5. Discuss alternative explanations, including small-state strategy, regional identity, professional respondent bias, and broader ASEAN norms.

 6. Correct language, grammar, and citation-formatting issues throughout the manuscript.

 Overall assessment

 Overall, this is a promising and relevant article that contributes to the literature on ASEAN, Brunei Darussalam, and foreign policy analysis by foregrounding public opinion in a context usually examined through elite and state-centric perspectives. It is suitable for indexing after revisions, especially if the authors clarify the methodology, moderate causal claims, and acknowledge the limitations of the survey data. My recommendation is approved with reservations.

Is the case presented with sufficient detail to be useful for teaching or other practitioners?

Yes

Is the work clearly and accurately presented and does it cite the current literature?

Partly

If applicable, is the statistical analysis and its interpretation appropriate?

Partly

Are all the source data underlying the results available to ensure full reproducibility?

Partly

Are the conclusions drawn adequately supported by the results?

Partly

Is the background of the case’s history and progression described in sufficient detail?

Yes

Reviewer Expertise:

Indonesian Foreign Policy, Diplomacy, ASEAN, conflict and cooperation issues in Southeast Asia.

I confirm that I have read this submission and believe that I have an appropriate level of expertise to confirm that it is of an acceptable scientific standard, however I have significant reservations, as outlined above.

# Response

**Published:** 2026-08-10

**Authors:** bama andika putra

**Affiliations:** University of Bristol School of Sociology Politics and International Studies, Bristol, England, UK

Comment 1:

 First, the main concern is the strength of the causal claim. The article shows alignment or consistency between public opinion and elite foreign policy, but the evidence consists mainly of cross-sectional survey data and interpretation of Brunei’s ASEAN policy. This does not fully demonstrate that elite views caused or shaped public opinion. The **authors should either soften causal language or provide clearer methodological justification**. **Terms such as “alignment,” “convergence,” and “consistency” are well supported,** **whereas “influenced by,” “shaped by,” or “formed by” require more direct evidence of transmission mechanisms**.

 We agree with Reviewer 2's statement. This article does not have the strength of the causal claim, and the safer option is to soften the causal language to avoid misinterpretations of the results. The revised paragraphs are below:

 Paragraph 7, section 1: To claim the consistency between the views of the Brunei elites and the public, this study utilizes the publicly available data from the ‘State of Southeast Asia 2025’ survey report, published by the ISEAS-Yusof Ishak Institute. In this case, the survey includes elites and the informed public, allowing this study to generalize arguments based on the views of those directly involved in the discourse on Brunei and China. The survey provides answers to how Brunei respondents view ASEAN in general, ASEAN’s role in contemporary regional challenges, questions on ASEAN’s potential alignment, and the way forward for the regional organization (Seah *et al.*, 2025). Using this dataset, this study is able to isolate how Brunei respondents view the regional organization in 2025 and connect this to the potential influence that Brunei elites had in shaping those preferences, based on Brunei’s ASEAN policies during and after its ASEAN chairmanship in 2021.

 Paragraph 3, section 2: In doing so, Boucher’s ‘top-down’ model serves as a good starting point to better understand the relations between the Brunei public and foreign policy decisions. As Boucher suggests, there is a clear gap between what political elites know and what the public understands (Boucher, 2024). Consequently, it is expected that public opinions would use the views of the elites as “[…] a heuristic shortcut for them (the public) to form an opinion” (Boucher, 2024, p.254). This point has been repeatedly argued by past scholars who fall under the broad category of the top-down model, believing that the interactive relationship between the two variables is best described as consistent with the elite’s views (Zaller, 1992; Druckman, 2001; Western, 2005; Boucher, 2024).

 Paragraph 4, section 3: Bridged to the context of Brunei’s public opinions in matters related to ASEAN, one can conclude that their embraced opinions are consistent with Brunei’s political elites’ view of the regional organization. As perhaps clear by this point, Sultan Hassanal Bolkiah has a favorable perception of ASEAN, and this is evident throughout Brunei’s ASEAN chairmanships. Therefore, the argument proposed is that Brunei’s past active engagements continue to effectively shape policy preferences and public views of Brunei’s external outlook in relation to the Southeast Asian region and ASEAN.

 Paragraph 7, section 2: Although existing scholarship documents an evolving discourse seeking to make sense of Brunei’s agency and active participation in ASEAN, the role of public opinion remains unexplored in these studies. Therefore, to better our understanding of how Brunei citizens perceive ASEAN’s importance (and related issues), this study zooms in on Brunei's perceptions of the regional organization and identifies potential variables that could influence these perceptions. Arguing specifically for a top-down model (alignment between public opinion and elite perceptions/foreign policy conduct), this study will be the first to examine how public opinion is formed in Brunei, measuring the interactive relationship between public opinion and regional foreign policy.

 Paragraph 9, section 4.1: The data presented in this section show several unique patterns in how Brunei’s public views ASEAN. Within the context of international developments, trust, and the strategic rivalries influencing the Southeast Asian region, there seems to be a strong convergence of views between the Brunei public and the Sultanate. As will be explored in the next section through Boucher’s top-down analytical framework, it will be shown that Brunei’s public opinion in 2025 converges with the Sultan’s strategic views on ASEAN, expectations, and future projections. This can be seen in the Sultanate’s recent policies, including during Brunei’s ASEAN chairmanship in 2021, which clearly reflected the Sultan’s priorities and views regarding ASEAN.

 Paragraph 2, section 5: In doing so, Boucher’s 2024 analytical framework of the ‘top-down’ model serves this study’s intentions well. In understanding the nexus between public opinion and foreign policy, Boucher’s study argues that elites’ views are consistent with the public's preferences and opinions. Utilizing data from the 2025 State of Southeast Asia survey report, this study bridges the analytical framework by means of identifying consistency between the Sultanate’s ASEAN policies and perceptions towards the regional organization (primarily during Brunei’s 2021 ASEAN chairmanship), with the public perceptions of Brunei respondents towards ASEAN (seen through respondents’ answers in the 2025 survey).

 Comment 2:

 Second, the representativeness of the survey data requires clearer discussion. The article notes that the 2025 survey had 2,023 respondents, with Brunei accounting for 7.4%, and that respondents were screened for ASEAN knowledge and interest in current affairs. This means that the data seems to reflect informed or policy-attentive respondents from academia, government, regional organizations, NGOs/media, and the private sector, rather than the entire Brunei population. **The authors should, therefore, qualify repeated references to “the Brunei public,” “Brunei people,” and “general public.”**

**Response: Thank you for this comment, which is much similar to the suggestion of Reviewer 1.. To clarify the methodology, we have revised several paragraphs that makes clear who the respondents were in the ISEAS survey, and why they were chosen. The first paragraph revised is the slight description in the methodology of the article, followed by small areas of revised terminologies throughout the article.**

 Paragraph 7, section 1: To claim the consistency between the views of the Brunei elites and the public, this study utilizes the publicly available data from the ‘State of Southeast Asia 2025’ survey report, published by the ISEAS-Yusof Ishak Institute. In this case, the survey includes elites and the informed public, allowing this study to generalize arguments based on the views of those directly involved in the discourse on Brunei and China. The survey provides answers to how Brunei respondents view ASEAN in general, ASEAN’s role in contemporary regional challenges, questions on ASEAN’s potential alignment, and the way forward for the regional organization (Seah *et al.*, 2025). Using this dataset, this study is able to isolate how Brunei respondents view the regional organization in 2025 and connect this to the potential influence that Brunei elites had in shaping those preferences, based on Brunei’s ASEAN policies during and after its ASEAN chairmanship in 2021. Important to note here is that the respondents in the ISEAS survey represent elements from academia, government, regional organizations, NGOs/media, as well as the private sector, which are more informed populations of Brunei (rather than the overall population).

 Paragraph 2, section 2: Nevertheless, alternative themes can be explored regarding Brunei and ASEAN. Sidelining for the moment the variable of public opinion, several minor discourses still relate to the discussions put forward in this study: Brunei’s placing of ASEAN as its foreign policy cornerstone, Brunei’s use of ASEAN to counter great power rivalries, as well as discussions specific to Brunei’s ASEAN chairmanships. Revealing how existing studies have interpreted and framed Brunei’s foreign policy in relation to ASEAN enables this study to identify potential variables that influence the Brunei respondent’s foreign policy perceptions.

 Paragraph 8, section 3: In doing so, this discussion section is structured as follows. First, it aims to reveal how the Bruneian people perceive the significance of ASEAN across different questions and elements related to Southeast Asian regional dynamics. Understanding Brunei’s public perceptions is done through the use of published data from the State of Southeast Asia survey report 2025, specifically focusing on Brunei respondents’ answers. Several questions focus on Brunei respondents’ perceptions of ASEAN amid international developments, how ASEAN responds to major powers’ regional influence, and ASEAN’s position in the US-China rivalry. After that, it offers a top-down model that focuses on the thesis that Brunei’s elites' foreign policy preferences are aligned with the perceptions of the Brunei respondents, primarily through events during Brunei’s ASEAN chairmanship in 2021. Elite perceptions, in the context of Brunei, are defined as the policies undertaken by the nation as a whole, without distinguishing between those of different ministries. As an absolute monarchy, the expectation is that differences of opinion are near non-existent, and that Sultan Hassanal Bolkiah is the driving force of how Brunei should act in relation to ASEAN.

 Paragraph 1, section 4: There is no doubt that Brunei elites view ASEAN with great importance; however, does a similar perception exist among the Brunei people, as represented by the informed public? This section first examines Brunei’s public perceptions, focusing on the dataset of Brunei respondents that this study retrieved from the ISEAS Yusof Ishak Institute’s State of Southeast Asia survey report 2025. After revealing those insights, the analytical framework of Boucher’s interactive relationship between public opinion and foreign policy is adopted to decipher what causes the Brunei respondents to embrace certain perceptions of ASEAN.

 Paragraph 2, section 4.1: The Brunei respondents’ views on ASEAN can be divided into several specific sectors. The first sector asks the Brunei respondents for their opinions on certain international developments related to ASEAN. When asked, “what is your top concern about ASEAN,” as seen in Table 1 below, Brunei respondents mainly expressed concerns over the regional organization itself lacking the capacity to be relevant in the new world order (30.9%), followed by an external-based challenge that relates to ASEAN being an “[…] arena of major power competition and its member states may become major power proxies” (26.2%) (Seah et al., 2025, p.16). Meanwhile, an equal number of votes chose the concerns of disunity (13.4%), disconnection from ordinary people (16.8%), and the inability to return to pre-pandemic economic growth rates (12.8%). In relation to that question, when asked how ASEAN can enhance its resilience, the majority of Brunei respondents selected accelerating measures for intra-regional trade and investment (28.2%), followed by the need to cooperate with strategic partners (24.8%) (Seah *et al.*, 2025).

 Paragraph 1, section 4.2: To argue that a top-down model reflects the interactive relationship between public opinion and foreign policy, the elite’s views and foreign policy must be highlighted. Therefore, in alignment with the analytical framework of Boucher’s 2024 study, this section will explore the preferences and voices of the Brunei respondents as expressed in the State of Southeast Asia survey report 2025 and argue for consistency between the Brunei respondents’ perceptions and the Sultan’s. However, it also claims there could be room for a combination of a top-down and bottom-up model in Brunei, albeit in a limited sense.

 Paragraph 2, section 4.2: One of the first questions asked of Brunei respondents was what they perceived as the main concerns they had regarding ASEAN. Unsurprisingly, the majority of respondents expressed concern about great power rivalries and the need for ASEAN to enhance its resilience. Nevertheless, on the question of how ASEAN should strengthen its resilience, Brunei respondents voted for ASEAN to focus on increasing intra-regional trade and investment. One way of interpreting this view is the Sultan’s introduction of *Wawasan Brunei 2035*. The grand strategy acknowledges that for too long, Brunei Darussalam has been dependent on its hydrocarbon resources (Slesman and Baha, 2023; Teja, 2024; CEIC, 2025). Therefore, moving forward, the Sultan is seeking ways to develop sustainable economic measures and diversify the economy (CSPS, 2022; Koh, 2024; Jetin and Khalid, 2025), a vision eventually embraced by the general public of Brunei. However, as a small nation of approximately half a million people, Brunei has severely limited choices. Given the Brunei respondents' embrace of increased intra-regional trade, there is therefore a certain level of consistency between the Brunei respondent’s voices and the Sultan's approach to diversifying the state’s economy.

 Paragraph 4, section 4.2: The Sultan’s South China Sea in the past two decades can also shed light on the Brunei respondents’ perception of how ASEAN should respond to the South China Sea. At first, the results of the 2025 State of Southeast Asia survey may suggest that Bruneians are encouraging a more decisive response from ASEAN. However, a deeper look at the available options would show that the six options themselves lean towards decisive action, albeit in different forms (excluding the option of ASEAN doing nothing) (Seah *et al.*, 2025). Consequently, the Brunei respondent’s choice to push ASEAN to support actions that deter aggressive behavior and uphold international law remains consistent with the Sultanate's eventual policies.

 Paragraph 6, section 4.2: Similarly, the respondents also expressed confidence in the CoC. This is indeed in alignment with the discourses the Sultanate has disseminated over the past two decades regarding the disputed waters of the South China Sea (Espena and Uy, 2020; IMOA, 2020; Putra, 2024; Sands, 2026). Brunei believes that it is essential to keep the momentum to conclude the CoC, as it is one of the platforms that have the potential to counter the challenges encountered by claimant states in the South China Sea, and allows for energy exploration deals to take place as a means to reflect the cooperative nature of the seas (CGTN, 2018; Druce and Julay, 2019; Yilmaz, 2025).

 Paragraph 7, section 4.2: Moving on to the topic of major powers’ regional influence in Southeast Asia, there is also a strong alignment between the views held by the respondents and the actual foreign policy pursued by Brunei years earlier. One perception that may surprise is that the respondents chose China and the US as the strongest political and strategic influences in Southeast Asia, ahead of ASEAN (in third place). Nevertheless, one needs to look more closely at recent trends in Brunei’s bilateral relations with the two countries to understand why the the respondents embraces such a perspective.

 Paragraph 10, section 4.2: Lastly, one can also draw on the interactive relationship between the respondent’s opinions and the elite’s decided policies and perceptions, based on how the public perceives where ASEAN should position itself amid the US-China strategic rivalry in Southeast Asia. As seen in the 2025 survey report, the majority of respondents chose the option that ASEAN should enhance its capacity to counter pressures from the major powers. The second option was that Brunei respondents chose that ASEAN should not side with China or the US. In the responses, another consistency emerges that aligns with the top-down analytical framework. Some arguments state that Brunei is hedging with the US and China (simultaneously), which shows that Brunei does not wish to side with one power to counter the other (Husseini, 2023). As the Sultan’s position during the 2021 Brunei chairmanship of ASEAN, the critical part for ASEAN is to enhance its capabilities and make it relevant to Asia Pacific geopolitics through measures as stipulated in the ASEAN Outlook on the Indo-Pacific (Acharya, 2019; ASEAN, 2019; Singh and Tsjeng, 2020).

 Paragraph 3, section 5: This study reveals the public opinions of the Brunei people and shows a strong alignment between the Sultan’s past ASEAN perspectives and approaches and the actual perceptions embraced by the general public. Regarding how Brunei respondents perceived several international developments, for example, Brunei identified that one area it should focus on is intra-regional trade and investment. This means that correlations are made between the Sultan’s recent efforts to diversify Brunei’s economy and how they perceive areas where ASEAN can benefit from further expansion. Furthermore, the Sultan’s 2021 engagements with Myanmar, which led to the eventual ‘Five-Point Consensus’ in the same year, have also been embraced in public perceptions, which called for the establishment of active dialogue with all relevant stakeholders in Myanmar's internal conflicts, including the NUG.

 Paragraph 5, section 5: Meanwhile, when Brunei respondents were asked how they viewed the major power contestations in Southeast Asia, they expressed trust in ASEAN and a slight alignment with China. In the question of political and strategic influence, and the actor trusted to champion the global free trade agenda, China claimed the first position from the perspective of the respondents. This is consistent with the Sultan’s foreign policy over the past decade, in which the long-term grand strategy of diversifying the economy has received a positive response from Chinese officials, leading to the convergence of *Wawasan Brunei 2035 *and the Belt and Road Initiative. Nevertheless, the importance of ASEAN has remained relevant in both inquiries, ranking significantly higher than those of the other major powers. When respondents were asked what ASEAN should do vis-à-vis the US-China quest for influence, the respondents chose to focus on the within, by enhancing ASEAN’s resilience and unity to counter any pressure from the main powers. This is again consistent with the Sultanate’s foreign policy, which resembles hedging with the US and China, as well as continuing to support ASEAN-introduced measures that would enhance the regional organization's capacity to address uncertainties to come.

 Paragraph 6, section 5: Nevertheless, the analytical framework used to assess Brunei’s foreign policy preferences here comes with its limitations. The most prominent is that the survey reports considered take the views of screened elites and the informed public. Therefore, it does not have the capacity to generalize the overall views of the Brunei public, as it can only argue on the basis of alignment and consistency of views between the respondents who are Bruneian nationals and elites.

 Comment 3:

 Third, the methodology should be strengthened. The article describes itself as qualitative, yet much of the analysis relies on descriptive survey percentages. **The authors should clarify whether it is a qualitative case study using secondary survey data** or a descriptive interpretive analysis. **They should also explain how survey questions were selected, how elite views were operationalized, and how policy documents, speeches, or ASEAN chairmanship actions were chosen and interpreted**.

 The methodology section has been revised to address several of the concerns raised by Reviewer 2. The revisions:

 Abstract: Methods: To reveal Brunei's perceptions vis-à-vis ASEAN and explore the relationship between those views and the Sultanate’s ASEAN policies, this qualitative case study examines the applicability of Boucher’s 2024 analytical framework of the ‘top-down’ approach, which argues that the people eventually embrace elites’ views on foreign policy. It utilizes the secondary survey data published in the State of Southeast Asia 2025.

 Paragraph 7, section 1: To claim the consistency between the views of the Brunei elites and the public, this study utilizes the publicly available data from the ‘State of Southeast Asia 2025’ survey report, published by the ISEAS-Yusof Ishak Institute. In this case, the survey includes elites and the informed public, allowing this study to generalize arguments based on the views of those directly involved in the discourse on Brunei and China. The survey provides answers to how Brunei respondents view ASEAN in general, ASEAN’s role in contemporary regional challenges, questions on ASEAN’s potential alignment, and the way forward for the regional organization (Seah *et al.*, 2025). Using this dataset, this study is able to isolate how Brunei respondents view the regional organization in 2025 and connect this to the potential influence that Brunei elites had in shaping those preferences, based on Brunei’s ASEAN policies during and after its ASEAN chairmanship in 2021. Important to note here is that the respondents in the ISEAS survey represent elements from academia, government, regional organizations, NGOs/media, as well as the private sector, which are more informed populations of Brunei (rather than the overall population). Furthermore, to argue the alignment of views between Brunei’s informed public and the Sultan, policy documents of Brunei, speeches, and general indication of alignment preferences through primary documents are considered in this study.

 Comment 4:

 Fourth, the article **should more clearly distinguish correlation, interpretation, and evidence. For example, linking respondents’ preference for intra-regional trade and investment to Wawasan Brunei 2035 is plausible, but the article does not directly show that respondents knew or were influenced by that vision.** Similarly, claims that public views on Myanmar or the South China Sea were shaped by **Brunei’s 2021 ASEAN chairmanship would be stronger with evidence from public discourse, media framing, government communication, speeches, education/socialization, or longitudinal comparison before and after 2021.**

**We agree that there is room to improve the point of this study’s correlation, interpretation and evidence. However, I do not engage with this recommendation due to several reasons. First, to adopt a longitudinal comparison between the 2024 survey and those before, this would go beyond the reach of this article’s case study, which is primarily focused on 2024 due to the unique context during that time. Furthermore, the 2021 ASEAN chairmanship already provides a firm indication of Brunei’s alignment preferences, which does not require this study to delve deeper into the specifics of how it is being framed. Third, using public discourse and media framing analysis would be difficult within the context of Brunei, considering the highly restricted information flow of the country. Therefore, the discourses introduced by the Sultanate is straightforward, and hard to misinterpret, despite the lack of a clear method to analyze the emerging discourses.**

 Comment 5:

 Fifth, alternative explanations should be addressed. **Support for ASEAN or preference for hedging may also reflect Brunei’s small-state vulnerability, economic dependence, regional identity, media environment, education, respondents’ professional background, or broader ASEAN norm**s. The article briefly mentions an interactive relationship between public opinion and elites, but this point remains underdeveloped.

 We agree with the suggestion of the article. Important to note, however, the primary burden of this case study is to argue the potential relevance of the top-down approach in making sense of the alignment between the Brunei informed public and the Sultan’s policy preferences. Therefore, I acknowledge the presence of these alternative explanations, and cite that these can be explored in future studies. The revisions at the end of the conclusion section:

 Nevertheless, the analytical framework used to assess Brunei’s foreign policy preferences here comes with its limitations. The most prominent is that the survey reports considered take the views of screened elites and the informed public. Therefore, it does not have the capacity to generalize the overall views of the Brunei public, as it can only argue on the basis of alignment and consistency of views between the respondents who are Bruneian nationals and elites. Second, it also acknowledges that there is room to interpret that the Sultanate’s foreign policy directions are influenced by other factors that are well elaborated within the small state, economic dependence, regional identity, media environment, and regionalization norm literature. However, it is beyond the scope of this article to argue each and every alternative explanation, primarily as this article falls under the category of a case study. These explanations could be an intriguing point of inquiry in future studies.

 Comment 6:

 Sixth, the data availability statement is useful because it links to the 2024 and 2025 State of Southeast Asia survey reports. However, the **authors should clarify whether they used only public aggregate tables or country-level raw data. If aggregate percentages were used, this should be stated; if raw data were used, th**e authors should explain where the dataset can be accessed and how the Brunei subset was isolated.

 We have revised the data availability statement below:

**Data Availability Statement**: The data used for this study are aggregated percentages published by the ISEAS-Yusof Ishak State of Southeast Asia survey reports, with specific percentages on the informed public of Brunei being traceable due to the survey’s country focus approach. These documents can be accessed online:

 1. The State of Southeast Asia 2024 Survey Report (Seah et al., 2024): https://www.iseas.edu.sg/centres/asean-studies-centre/state-of-southeast-asia-survey/the-state-of-southeast-asia-2024-survey-report/

 2. The State of Southeast Asia 2025 Survey Report (Seah et al., 2025): https://www.iseas.edu.sg/centres/asean-studies-centre/state-of-southeast-asia-survey/the-state-of-southeast-asia-2025-survey-report/ 

 Comment 7:

 Finally, several presentation issues should be corrected. **For example, “Brunei respondent’s perceptions” should be “Brunei respondents’ perceptions”; “bottom-down model” appears where “bottom-up model” seems intended; and “soroka wlezien 2004, sobel 2001” is inconsistently formatted. **Careful proofreading is needed to improve grammar, consistency, and reference formatting.

# Reviewer report: Reviewer response for version 1

**DOI:** 10.5256/f1000research.198599.r482840

**Published:** 2026-05-13

**Authors:** Kittisak Wongmahesak

**Affiliations:** 1 North Bangkok University, Bangkok, Bangkok, Thailand

While the article is well-written, it must address the following to enhance its scientific rigor:

 1) Theoretical Nuance: The 'top-down' model needs to explain the mechanisms of information flow in Brunei's controlled media environment to avoid being deterministic.

 2) Institutional & Legal Depth: The authors should move beyond survey percentages by analyzing the structural constraints of the ASEAN Charter. Specifically:

 - On Myanmar: Use Ref 1 - Limsiritong (2018) and Ref 2 - Limsiritong & Sookhakich (2023) to discuss the legal limitations of AICHR and Charter breaches, and contextualize the Sultan’s diplomatic choices.

 - On South China Sea: Incorporate Ref 3 - Limsiritong et al. (2019) to explain why ASEAN’s decision-making modes often struggle to meet the 'principled stance' desired by the public.

 - On Economic Trust: Integrate Ref 4 - Sookhakich (2019) to provide a critical view of the legal hurdles in the ACFTA, balancing the public’s high trust in China with the realities of dispute settlement.

 3) Methodological Transparency: Explicitly state the limitation that ISEAS survey respondents are 'screened' elites/informed public, which inherently biases results toward top-down alignment.

 Below is the specific guidance on where the authors should incorporate these sources to strengthen their manuscript:

 - In Section 4.2.1 (Origins and Evolution of Boundary Conflicts/Myanmar issue): Authors should incorporate Ref 1 - Limsiritong (2018) and Ref 2 - Limsiritong & Sookhakich (2023). These sources provide a critical legal perspective on the AICHR's power limitations and the "serious breach" of the ASEAN Charter regarding the Myanmar coup. This will help explain why the Sultan’s "Five-Point Consensus" was chosen as the diplomatic path despite its perceived slow progress.

 - In Section 4.2.2 (South China Sea discussion): Authors should cite Ref 3 - Limsiritong et al. (2019). This reference is essential to explain the structural difficulties of ASEAN decision-making from a Charter perspective, offering a necessary counterweight to the public’s desire for a "principled stance."

 - In Section 4.2.3 (Economic alignment/China): Authors should integrate Ref 4 - Sookhakich (2019). This source highlights the legal hurdles and limits of dispute settlement under the ACFTA framework, offering a more balanced view of the "trust" the Brunei public places in China's economic leadership.

Is the case presented with sufficient detail to be useful for teaching or other practitioners?

Yes

Is the work clearly and accurately presented and does it cite the current literature?

Partly

If applicable, is the statistical analysis and its interpretation appropriate?

Yes

Are all the source data underlying the results available to ensure full reproducibility?

Yes

Are the conclusions drawn adequately supported by the results?

Yes

Is the background of the case’s history and progression described in sufficient detail?

Yes

Reviewer Expertise:

Political Science, Public & Private Management, International Political Economy, Social Research

I confirm that I have read this submission and believe that I have an appropriate level of expertise to confirm that it is of an acceptable scientific standard, however I have significant reservations, as outlined above.

## References

[1] : The Power Limitation of ASEAN Inter-Governmental Human Rights Commission Under the Perspective of ASEAN Charter in Case of the Rohingya Issue. *SSRN Electronic Journal* .2018; 10.2139/ssrn.3318102 10.2139/ssrn.3318102

[2] : Is There a Serious Breach of ASEAN Charter?: A Case Study of the Myanmar Military Coup 2021 Violence Against Civilians. *SSRN Electronic Journal* .2023; 10.2139/ssrn.4540043 10.2139/ssrn.4540043

[3] : The Difficulty of ASEAN Decision Making Mode on South China Sea Dispute: The ASEAN Charter Perspective. *SSRN Electronic Journal* .2019; 10.2139/ssrn.3459315 10.2139/ssrn.3459315

[4] : Limits and Challenging Factors of the Dispute Settlement Mechanism in the Light of China- ASEAN Free Trade Agreement Perspective. *SSRN Electronic Journal* .2019; 10.2139/ssrn.3459317 10.2139/ssrn.3459317

# Response

**Published:** 2026-08-10

**Authors:** bama andika putra

**Affiliations:** University of Bristol School of Sociology Politics and International Studies, Bristol, England, UK

Comment 1:

 1) Theoretical Nuance: The 'top-down' model needs to explain the mechanisms of information flow in Brunei's controlled media environment to avoid being deterministic.

 : We agree that the ‘top-down model’ requires further elaboration. In section 3 (analytical framework), we have revised it to make clear what the model argues. First, it shows that the causality is probabilistic, not the sole determinant of the foreign policy behavior. Second, I clarify the nature of the information flow in Brunei, which is expected to lead to the embracement of certain views that align with the Sultan. The revised paragraphs:

 Paragraph 4, section 3: Bridged to the context of Brunei’s public opinions in matters related to ASEAN, one can conclude that their embraced opinions are consistent with Brunei’s political elites’ view of the regional organization. As perhaps clear by this point, Sultan Hassanal Bolkiah has a favorable perception of ASEAN, and this is evident throughout Brunei’s ASEAN chairmanships. Therefore, the argument proposed is that Brunei’s past active engagements continue to effectively shape policy preferences and public views of Brunei’s external outlook in relation to the Southeast Asian region and ASEAN. 

 Paragraph 6, section 3: Therefore, it is beyond the scope of this study to assert which model would be most favorable for explaining the relationship between public opinion and foreign policy in the context of Brunei vis-à-vis ASEAN. The discussions, therefore, will first focus on explaining how the top-down model is most likely the one that allows the public to embrace certain perceptions. However, it will also acknowledge the potential for citizens’ views to influence the Brunei elite’s perceptions of ASEAN. The arguments presented in this article also consider the highly restricted nature of Brunei’s information flow, in which accessible information would tend to be based on the Sultan’s preferences. 

 Comment 2:

 2) Institutional & Legal Depth: The authors should move beyond survey percentages by analyzing the structural constraints of the ASEAN Charter. Specifically:

 - On Myanmar: Use Ref 1 - Limsiritong (2018) and Ref 2 - Limsiritong & Sookhakich (2023) to discuss the legal limitations of AICHR and Charter breaches, and contextualize the Sultan’s diplomatic choices.

 - On South China Sea: Incorporate Ref 3 - Limsiritong et al. (2019) to explain why ASEAN’s decision-making modes often struggle to meet the 'principled stance' desired by the public.

 - On Economic Trust: Integrate Ref 4 - Sookhakich (2019) to provide a critical view of the legal hurdles in the ACFTA, balancing the public’s high trust in China with the realities of dispute settlement.

 Response: Thank you for the recommendations. We have incorporated all of the recommended literature, as they all have strong correlations to the discussions that I am bringing forward in the article. The revised paragraphs:

 Paragraph 3, section 4.2: Specifically looking at the Brunei respondents’ views on the tensions in Myanmar, what can explain the respondents’ preferences to engage with all actors in Myanmar? Back in 2021, during Brunei’s ASEAN chair, Brunei’s role was pivotal in the conclusion of the ‘Five-Point Consensus.’ The consensus reflected ASEAN’s interests in responding to the Myanmar crisis, calling for an immediate end to violence, acceptance of humanitarian aid, dialogue with all involved conflicting parties, and allowing the ASEAN envoy to visit Myanmar (Thuzar and Seah, 2022; APHR, 2023; Aung, 2023; Tene, 2024). Sultan Hassanal Bolkiah, at that time, also expressed his willingness to mediate among all parties concerned, which was welcomed by the NUG (Bandial, 2021). Therefore, when asked four years later about their opinion on how to respond to the Myanmar crisis, the Brunei respondents aligned on the need to engage the NUG to facilitate diverse interests and views in working toward a solution to the situation. Within the same question, only a slight minority answered that ASEAN should not do anything (4.7%) (Seah et al., 2025), which further shows consistency between the public’s opinions in 2025 being formed by the Sultan’s ASEAN approach and policies back in 2021. The unique position embraced by the Sultan is related to the vast discourses on issues related to ASEAN’s power limitations, especially within the context of human rights in Southeast Asia (Limsiritong, 2019; Limsiritong and Sookhakich, 2023). 

 Paragraph 5, section 4.2: 

 As argued by past scholars, for example, Brunei is known to have a somewhat muted response to the South China Sea (Putra, 2021, 2024; Kurniati, Laksono and Aulia, 2025; Sands, 2026). This is slightly confusing, considering that Brunei is indeed a claimant state in the South China Sea and that intrusions by Chinese law enforcement vessels have occurred in the past (Elleman, 2017; Hart, 2018; Jacques, 2018; Druce and Julay, 2019). Nevertheless, although this has been the common interpretation, we can also see that the Sultanate is balancing this approach with a more regionally oriented resolution towards the crisis, and one that is centered on the potential role of ASEAN in managing the South China Sea dispute (Espena and Uy, 2020; Hayat, 2021; IRSEA, 2021). As also argued in the past, ASEAN has the capacity to provide an alternative means to counter issues in the region, which is based on the ASEAN Way (Limsiritong, Springall and Rojanawanichkij, 2019). Therefore, although Brunei is a silent claimant state to the South China Sea, it still echoes the importance of maintaining the rule of law in the South China Sea, and undertakes joint exercises with the US as a means to counter potential aggressive behaviors taking place in the disputed waters (Wilson, 2024; Nguyen, 2025). Therefore, the results of the State of Southeast Asia survey, which reflect the Brunei people’s perceptions of the nexus between the disputed waters and ASEAN, are consistent with the stance held by the Sultanate. 

 Paragraph 8, section 4.2: With China, the convergence of interests between Brunei and China has been strong. This convergence is primarily marked by the alignment of Brunei's and China's grand strategies, namely Wawasan Brunei 2035 and the Belt and Road Initiative (Bo, 2017; Koh, 2024; Jetin and Khalid, 2025). The Sultanate has been open to allowing a vast number of Chinese foreign investments into Brunei, whether it be in the infrastructural construction dimensions, or in Brunei’s intentions of diversifying the economy through downstream industries of its oil and gas fields (CSPS, 2022; Slesman and Baha, 2023; Koh, 2024; Jetin and Khalid, 2025). China has risen in the ranks in terms of its importance to Brunei policymakers, as it is one of the few states openly willing to support the Sultan’s long-term vision. Therefore, it should be no surprise to see alignment between the Sultan’s policies and the eventual perception held by the Brunei public. As also seen in the question of the survey that asks the Brunei respondents’ confidence in the actor championing the global free trade agenda, the vast export-import cooperation between Brunei and China in the past decade (showing an upward trend) (WITS, 2025a, 2025b) also explains why respondents opted to choose China first, and ASEAN second. This is critical, considering that in cases such as the ASEAN-China Free Trade Agreement, it is noted that there are legal hurdles and limits to dispute settlement between ASEAN member states and China (Sookhakich, 2019). 

 Comment 3:

 3) Methodological Transparency: Explicitly state the limitation that ISEAS survey respondents are 'screened' elites/informed public, which inherently biases results toward top-down alignment.

 We agree with this suggestion. To enhance methodological transparency, we have revised two paragraphs within the document. The first is in the introduction section, and the second is in the conclusions. The revisions:

 Paragraph 7, section 1: To claim that the elites shape Brunei’s public opinions, this study utilizes the publicly available data from the ‘State of Southeast Asia 2025’ survey report, published by the ISEAS-Yusof Ishak Institute. In this case, the survey includes elites and the informed public, allowing this study to generalize arguments based on the views of those directly involved in the discourse on Brunei and China. The survey provides answers to how Brunei respondents view ASEAN in general, ASEAN’s role in contemporary regional challenges, questions on ASEAN’s potential alignment, and the way forward for the regional organization (Seah et al., 2025). Using this dataset, this study is able to isolate how Brunei respondents view the regional organization in 2025 and connect this to the potential influence that Brunei elites had in shaping those preferences, based on Brunei’s ASEAN policies during and after its ASEAN chairmanship in 2021. 

 Paragraph 6, section 5: Nevertheless, the analytical framework used to assess Brunei’s foreign policy preferences here comes with its limitations. The most prominent is that the survey reports considered take the views of screened elites and the informed public. Therefore, it does not have the capacity to generalize the overall views of the Brunei public, as it can only argue on the basis of alignment and consistency of views between the Brunei public and elites.
